# K^+^ Channel Inhibition Differentially Regulates Migration of Intestinal Epithelial Cells in Inflamed vs. Non-Inflamed Conditions in a PI3K/Akt-Mediated Manner

**DOI:** 10.1371/journal.pone.0147736

**Published:** 2016-01-29

**Authors:** Sebastian Zundler, Massimiliano Caioni, Martina Müller, Ulrike Strauch, Claudia Kunst, Gisela Woelfel

**Affiliations:** 1 Department of Internal Medicine I, Regensburg University Medical Center, Regensburg, Germany; 2 Department of Medicine 1, University of Erlangen-Nuremberg, Kussmaul Campus for Medical Research & Translational Research Center, Erlangen, Germany; NCMLS, Radboud University Nijmegen Medical Center, NETHERLANDS

## Abstract

**Background:**

Potassium channels have been shown to determine wound healing in different tissues, but their role in intestinal epithelial restitution–the rapid closure of superficial wounds by intestinal epithelial cells (IEC)–remains unclear.

**Methods:**

In this study, the regulation of IEC migration by potassium channel modulation was explored with and without additional epidermal growth factor (EGF) under baseline and interferon-γ (IFN-γ)-pretreated conditions in scratch assays and Boyden chamber assays using the intestinal epithelial cell lines IEC-18 and HT-29. To identify possibly involved subcellular pathways, Western Blot (WB)-analysis of ERK and Akt phosphorylation was conducted and PI3K and ERK inhibitors were used in scratch assays. Furthermore, mRNA-levels of the potassium channel KCNN4 were determined in IEC from patients suffering from inflammatory bowel diseases (IBD).

**Results:**

Inhibition of Ca^2+^-dependent potassium channels significantly increased intestinal epithelial restitution, which could not be further promoted by additional EGF. In contrast, inhibition of KCNN4 after pretreatment with IFN-γ led to decreased or unaffected migration. This effect was abolished by EGF. Changes in Akt, but not in ERK phosphorylation strongly correlated with these findings and PI3K but not ERK inhibition abrogated the effect of KCNN4 inhibition. Levels of KCNN4 mRNA were higher in samples from IBD patients compared with controls.

**Conclusions:**

Taken together, we demonstrate that inhibition of KCNN4 differentially regulates IEC migration in IFN-γ-pretreated *vs*. non pretreated conditions. Moreover, our data propose that the PI3K signaling cascade is responsible for this differential regulation. Therefore, we present a cellular model that contributes new aspects to epithelial barrier dysfunction in chronic intestinal inflammation, resulting in propagation of inflammation and symptoms like ulcers or diarrhea.

## Introduction

A single layer of epithelial cells is lining the surface of the gastrointestinal tract. It plays an indispensable role in the transport of nutrients, ions and water, but also exerts barrier function against potentially harmful agents in the lumen, e.g. bacteria or toxic dietary products. Access of such antigens through the intestinal epithelium can result in initiation of inflammatory cascades. Thus, preserving the integrity of the barrier is essential.

Superficial lesions of the IEC layer–as they occur in infection or inflammation–are quickly restored by a mechanism involving rapid restitution of the wound through dedifferentiation and migration of adjacent cells to the denuded area, followed by their proliferation and redifferentiation [1], [2].

It has been shown that this process of intestinal epithelial wound healing is promoted by a number of growth factors. For example, EGF upregulates epithelial migration in intestinal [3] and colonic cells [4] and thereby also quickly reduces damage of the epithelium as assessed by transepithelial resistance [5]. FGF similarly influences intestinal epithelial restitution and acts in a TGF-β-dependent manner [6]. TGF-β itself seems to selectively promote migration and not proliferation of IEC [7] and mediates the influence of other agents regulating wound healing of IEC [8]. Important regulatory intracellular pathways involved in the effects of these stimuli include the NF-κB-, PI3K- and ERK-cascades: After *in vitro* wounding of IEC, NF-κB is activated in an EGFR-dependent manner and its blockade leads to inhibition of restitution [9]. PI3K phosphorylation has been shown to be essential for wound healing *in vitro* by mediating downstream GSK3β phosphorylation [10]. ERK activation occurs rapidly after mechanical wounding of IEC [11] and is needed for chemokine receptor-dependent promotion of restitution [12].

According to findings in multiple cell lines, potassium channels also contribute to the regulation of cell migration and epithelial wound healing [13]. One study reported a promotion of IEC wound healing by blockade of potassium channels [14]. Blocking of KCNN4 in lung dendritic cells leads to a decreased response to chemotactic stimuli [15]. Alveolar epithelial repair after mechanical wounding is also mediated by potassium channels, which interact with EGF [16]. Investigations on potassium channels in rabbit corneal epithelial cell proliferation similarly revealed an interaction with EGF [17]. Despite these findings, little is known about the exact role of potassium channels and their interplay with growth factor-dependent signaling cascades in intestinal epithelial restitution.

Intestinal epithelial wound healing is of special interest as epithelial barrier dysfunction in genetically predisposed patients is believed to be one key element of the pathogenesis of IBD like Crohn's disease (CD) and ulcerative colitis (UC), although their precise etiology remains unclear. Factors contributing to barrier dysfunction include disruption of paracellular tight junctions [18], reduced secretion of antimicrobial peptides [19] and loss of epithelial cells, thereby leading to uncontrolled bacterial translocation [20].

CD is characterized by a predominant T helper 1 (Th1)-immune response, in which proinflammatory cytokines like IFN-γ, TNF-α and IL-12 play a central role. It has been demonstrated that IFN-γ is elevated in the mucosa and serum in CD [21] and is a key factor in different murine models of colitis [22], [23]. It is therefore thought to be a critical cytokine contributing to inflammation in CD [24].

We have recently demonstrated that IFN-γ alters EGFR-downstream signaling [25]. Moreover, IFN-γ is able to impair other essential cellular functions such as ion transport and tight junction organization [26], [27]. However, we have an incomplete understanding of the role of IFN-γ in intestinal epithelial wound healing.

In the present study we aimed to characterize the impact of potassium channels on intestinal epithelial wound healing. We investigated a potential influence of IFN-γ and sought to identify possible downstream targets in IEC cell lines. We show that IEC migration is differentially regulated by KCNN4 after pretreatment with IFN-γ compared to not pretreated conditions. Moreover, we demonstrate that the PI3K-pathway could account for this finding. We thereby provide new insights into deregulation of epithelial cellular functions in chronic intestinal inflammation.

## Materials & Methods

### Culture of cell lines

The newborn rat non-transformed IEC line IEC-18 (ATCC CRL-1589; passages 25–55) was a kind gift of Dr. Thomas Karrasch, Regensburg, who purchased the cell line from ATCC (Manassas, VA, USA) in 2007. It is derived from ileal crypt cells [28]. IEC-18 were cultured in DMEM with 4.5 g/L glucose and L-glutamine (PAA, Pasching, Austria) augmented with 5% FCS (Sigma Aldrich, Steinheim, Germany), 100 U/mL penicillin, 100 μg/mL streptomycin (PAA) and 0.7 mMol bovine insulin (Sigma-Aldrich) in a humidified atmosphere at 37°C with 10% CO_2_ [29], [30]. Subcultivation of IEC-18 was carried out twice per week in a ratio of 1:6. The human IEC line HT-29 [31] (passages 7–12) was generously provided by Dr. Rocío Lopez-Posadas, Erlangen, in 2015. HT-29 were cultured in DMEM with 4.5 g/L glucose and pyruvate (Life technologies, Darmstadt, Germany) with 10% FCS, 1% penicillin/streptomycin and 1% amphotericin and were subdivided 1:10 once per week. Fully supplemented cell culture medium was routinely filtered through steriflip filters (Millipore, Billerica, MA).

### Scratch wound healing assays

For scratch assays of IEC-18, 400000 cells were seeded in one-well LabTek chamber slides (glass bottom; Fisher Scientific, Schwerte, Germany) 48 h prior to wounding and incubated with fully supplemented medium for the next 24 h until they had reached confluency. Cells were then starved in serum-reduced medium (1% FCS, cf. [10]) with (“inflammatory conditions”) or without (“baseline conditions”) 100 ng/mL (1000 U/mL) recombinant rat IFN-γ (PromoCell, Heidelberg, Germany) (cf. [25]). Another 24 h later, wounding was performed using a 10 μL micropipette tip as described elsewhere [10], [14], [16], [32]. To detach cells adhering to the wound edges the cell layer was carefully rinsed with PBS (PAA) before new serum-reduced medium with or without potassium channel modulators and/or 5 nM recombinant rat EGF (Peprotech, Hamburg, Germany) was added. The following potassium channel modulators were used in the indicated concentrations: 5 mM Barium^2+^, 100 nM Iberiotoxin (IbTx), 200 nM Charybdotoxin (ChTx), 10 μM Clotrimazole (Clt; all from Sigma-Aldrich), 600 μM 1-ethyl-2-benzimidazolinone (1-EBIO; Tocris Bioscience, Bristol, Great Britain) (cf. [14], [33], [34]). The latter two modulators were dissolved in DMSO (Sigma-Aldrich) leading to a final DMSO concentration of 0.25% v/v (Clt) and 1% v/v (1-EBIO), respectively, in experiments.

To determine the wound healing course, the chamber slide was placed into the prewarmed (37°C), humidified and equilibrated (5% CO_2_) incubator of an Axiovert microscope and serial images of a wound area between 120000 μm² and 200000 μm² were taken every 15 minutes for the following six hours at 200-fold magnification. Imaging was controlled by AxioVision software and done with Axiocam Mr5c (all from Zeiss MicroImaging, Göttingen, Germany).

Scratch-wound assays with HT-29 cells were performed in 12 well cell culture plates (Greiner Bio-One, Frickenhausen, Germany). Following 24h of starvation in serum-reduced medium with or without 100 ng/ml recombinant human IFN-γ (Peprotech), cell layers were wounded as described above, serum-reduced medium with or without potassium channel modulators and with or without recombinant human EGF (Peprotech) and/or 10 μM of the PI3K inhibitor Ly294002 or 10μM of the ERK inhibitor 3-(2-Aminoethyl)-5-((4-ethoxyphenyl)methylene)-2,4-thiazolidinedione-hydrochloride (both from Sigma-Aldrich) was added and images of the wounds were taken with inverted microscopes (Leica, Wetzlar, Germany) right after wounding (t = 0h) and after 6h.

Analyses of the wound healing experiments were performed using AxioVision and ImageJ (NIH, Bethesda, MD) software by measuring original and remaining wound areas. If necessary, contrast and/or brightness of the pictures were digitally optimized.

### Migration assays in modified Boyden chambers

Lower wells of a modified Boyden Chamber (Receptor Technologies, Royal Leamington Spa, Great Britain) were filled with 30.2 μL serum-reduced medium with or without 10 μM Clt and separated from upper wells by a polycarbonate membrane with 8 μm pores (Osmonics, Moers, Germany). For adjustment of pH the chamber was transferred into an incubator for 30 min.

Confluent IEC-18 were starved in serum-reduced medium for 24 h with or without 100 ng/mL IFN-γ, washed with PBS and collected with Trypsin/EDTA (PAA). After centrifugation cells were resuspended in serum-reduced medium at a concentration of 200000 cells/mL and 50 μL of the suspension were pipetted in the upper compartment of the prepared Boyden Chamber.

After a migration period of six hours, the upper surface of the membrane was washed from attached cells with PBS and cells having migrated to the lower surface were fixed and stained with a Hemacolor kit (Merck, Darmstadt, Germany).

For cell counting, membranes mounted on a slide were placed under a microscope (Leica) and migrated cells in the center of the imprint of each well were counted at 200-fold magnification.

### Western Blot analysis

IEC-18 were seeded in cell culture dishes (Corning, Corning, NY) and starved for 24 h with or without 100 ng/mL IFN-γ after they had reached confluency. Using a multichannel pipette (Eppendorf, Hamburg, Germany) 16 perpendicular wounds were scratched into the cell layer with 10 μL pipette tips. Like this, an estimated number of 20% of the total cells were within a range of 250 μm from the wound margins. Afterwards, fresh serum-reduced medium with or without 10 μM Clt or 600 μM 1-EBIO, each with or without 5 nM EGF was added. After 30, 120 and 480 minutes of incubation whole cell lysates of each constellation were prepared as follows: Dishes were placed on ice and cells were rinsed with ice-cold PBS. Cells were detached with a cell scraper (Corning) and suspended in ice-cold PBS. After centrifugation, cell pellets were lysed in lysis buffer as described previously [25] and protein concentration was measured with a bicinchoninic acid (BCA)-protein quantification assay (Sigma-Aldrich). Volumes containing 7.5 μg of protein each were further diluted with aqua dest. and 6 x Laemmli buffer, boiled for five minutes and mounted on a 4–12% polyacrylamide gel to resolve proteins by electrophoresis. Proteins were then transferred onto a nitrocellulose membrane filter paper. NuPAGE^®^ instruments and reagents were used according to the manufacturer's instructions (all from Invitrogen, Carlsbad, CA). Membranes were blocked with 5% BSA (Biomol, Hamburg, Germany) in Tris-buffered saline with 0.1% Tween 20 (Sigma-Aldrich) (TBST) overnight.

Afterwards, membranes were incubated with the primary antibody in 5% BSA in TBST for one hour and washed six times with TBST prior to one hour of incubation with an HRP-conjugated secondary antibody. Membranes were washed another six times and exposed to 5 mL enhanced chemiluminescence (ECL)-solution (2.5 mM luminol, 0.1 M Tris-HCl and 0.4 mM p-coumarin acid–all from Sigma-Aldrich–in aqua dest.) mixed with 30% 1.53 μL hydrogen peroxide (Merck) for five minutes. Immunoreactive proteins were detected and densitometrical analyses were carried out with Image Quant software (Molecular Dynamics, Sunnyvale, CA).

The following primary antibodies were used and dissolved as indicated by the manufacturer: anti-Akt, anti-phospho-Akt (Ser473), anti-p44/42 MAPK, anti-phospho-p44/42 MAPK (Thr202/ Tyr204) (all from Cell Signaling, Danvers, MA) and for loading control anti-β-actin (Millipore, Billerica, MA). Secondary antibodies were anti-rabbit-IgG and anti-mouse-IgG (both from Santa Cruz, Heidelberg, Germany). For multiple use, membranes were stripped up to three times using Re-blot-plus strong solution (Chemicon, Temecula, CA).

### RNA isolation and Real Time-PCR Analysis

Surgical specimens and endoscopic biopsies from IBD and control patients were collected after written informed consent. The procedure was approved by the Ethics Committee of the University of Regensburg (record number 00/14) and performed according to the Declaration of Helsinki. IEC were isolated by accurate mechanical detachment of the mucosa from the submucosal layer with a dissecting set. The mucosa was then subjected to several washing steps and manual agitation after treatment with 2 mM EDTA (Carl Roth, Karlsruhe, Germany).

Following the manufacturer's instructions, RNA was isolated and purified from cells with the RNeasy Kit (Qiagen, Hilden, Germany). By measuring absorbance at 260 nm and 280 nm RNA concentration and purity was determined. cDNA was synthesized using Affinity script reagents (Agilent, Böblingen, Germany) along the manufacturer's recommendations.

Real-Time PCR was performed in triplicates for each sample with Brilliant II Mastermix for qPCR with high ROX (Agilent) in an AbiPrism^®^ Sequence Detector with SDS software (both from Applied Biosystems, Carlsbad, CA).

Primer sequences were as follows: human KCNN4 forward 5'-CCC TCA TCA AAA ACA CTC TCA CTA TG-3', reverse 5'-TCC AGT CGC CTG CAC TTG-3', probe 5'-FAM—TGC TAT GGA CGA CCT CCA GCT CTC AGT T—TAMRA-3', human KCNQ1 forward 5'-CGC ATG GAG GTG CTA TGC T-3', reverse 5'-GGC CTT CCG GAT GTA GAT CTT-3', probe 5'-FAM—AGA ACC CCG ACT CCT CCA CCT–TAMRA-3' (all from Eurofins MWG Operon, Ebersberg, Germany). Human GAPDH mix was used as endogenous control (Applied Biosystems, Carlsbad, CA). PCR conditions were two minutes of incubation at 50°C, followed by ten minutes at 95°C and 40 cycles of 15 seconds at 95°C for denaturation and 60 seconds at 60°C for annealing and extension each. Analysis of the results was performed by the ddCT method [35].

### Statistical analysis

Results of scratch wounding experiments and Boyden chamber assays are presented in scatter plots showing individual data points and mean with SEM. WB results are given as x-fold expression in relation to unwounded controls. Data from real-time PCR are shown as x-fold expression relative to the mean of the control group. Graphs were produced with GraphPad Prism (GraphPad Software, La Jolla, CA). Statistical comparison of groups was also performed with GraphPad Prism using ANOVA with appropriate post hoc tests or two-tailed student’s t-test where applicable. P-values < 0.05 were considered significant. Significance levels displayed in the graphs are * p < 0.05, ** p < 0.01, *** p < 0.001.

## Results

### Restitution of scratch-wounded IEC-18 monolayers is marked by migration of adjacent cells

In order to explore the mechanisms involved in gastrointestinal epithelial wound healing and to investigate a possible contribution of potassium channels to its regulation, we examined wound healing responses of IEC lines after mechanical injury.

**Fig 1:**
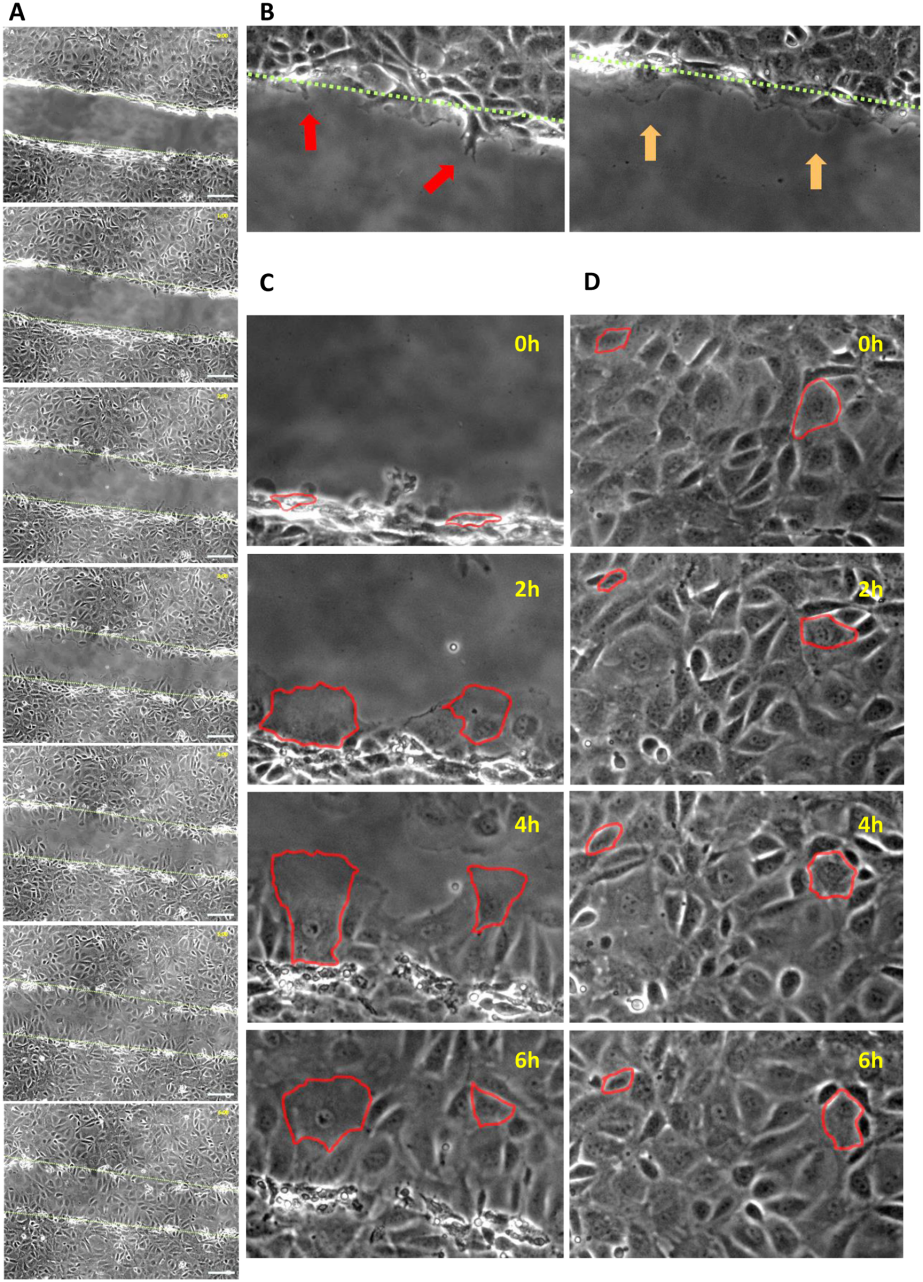
Restitution of scratch-wounded IEC-18 monolayers is marked by migration of adjacent cells. A: representative sequence of images taken every hour (from top to bottom) beginning directly after mechanical wounding of a confluent IEC-18 monolayer. The approximate location of the initial wound margin (top picture) is indicated by a green dashed line and also displayed in subsequent images. Scale bar: 100μm. B: Representative images of migrating cells sending ahead filopodia (red arrows) and pseudopodia (orange arrows). C: Exemplary sequence of processes at the wound margin. Two adjacent cells are edged in red and shown at the indicated time points. Over time they migrate into the denuded area undergoing considerable morphological changes. D: Exemplary sequence of events within the cell sheet distant from the wound. Two representative cells are edged in red and show only bare movements or changes in shape over six hours.

It is well known that migration is a cornerstone of intestinal epithelial restitution [2]. In an attempt to demonstrate this process, IEC-18 monolayers were wounded and serially imaged for six hours. As expected, we observed that cells in close neighborhood of the wound closed the gap by collectively migrating into the denuded area and undergoing considerable morphological changes including the protrusion of filopodia and pseudopodia (Fig 1A–1C; S1 Video). To the contrary, distant cells did barely move or change their shape and were thus not implicated in wound closure (Fig 1D; S1 Video). Moreover, cell proliferation did not relevantly contribute to epithelial restitution as the absolute number of cells only marginally changed within this timeframe (S1A Fig). This was confirmed by cell density measurements, which revealed that the cell density at the wound margin decreased over the course of the experiments due to cell migration with accompanying increase in cell size, while the cell density at distant locations remained approximately constant (S1B Fig). Hence, corresponding to established models [2], proliferation seems to be dispensable for early stages of restitution.

### Inhibition of Ca^2+^-dependent potassium channels enhances wound healing

**Fig 2:**
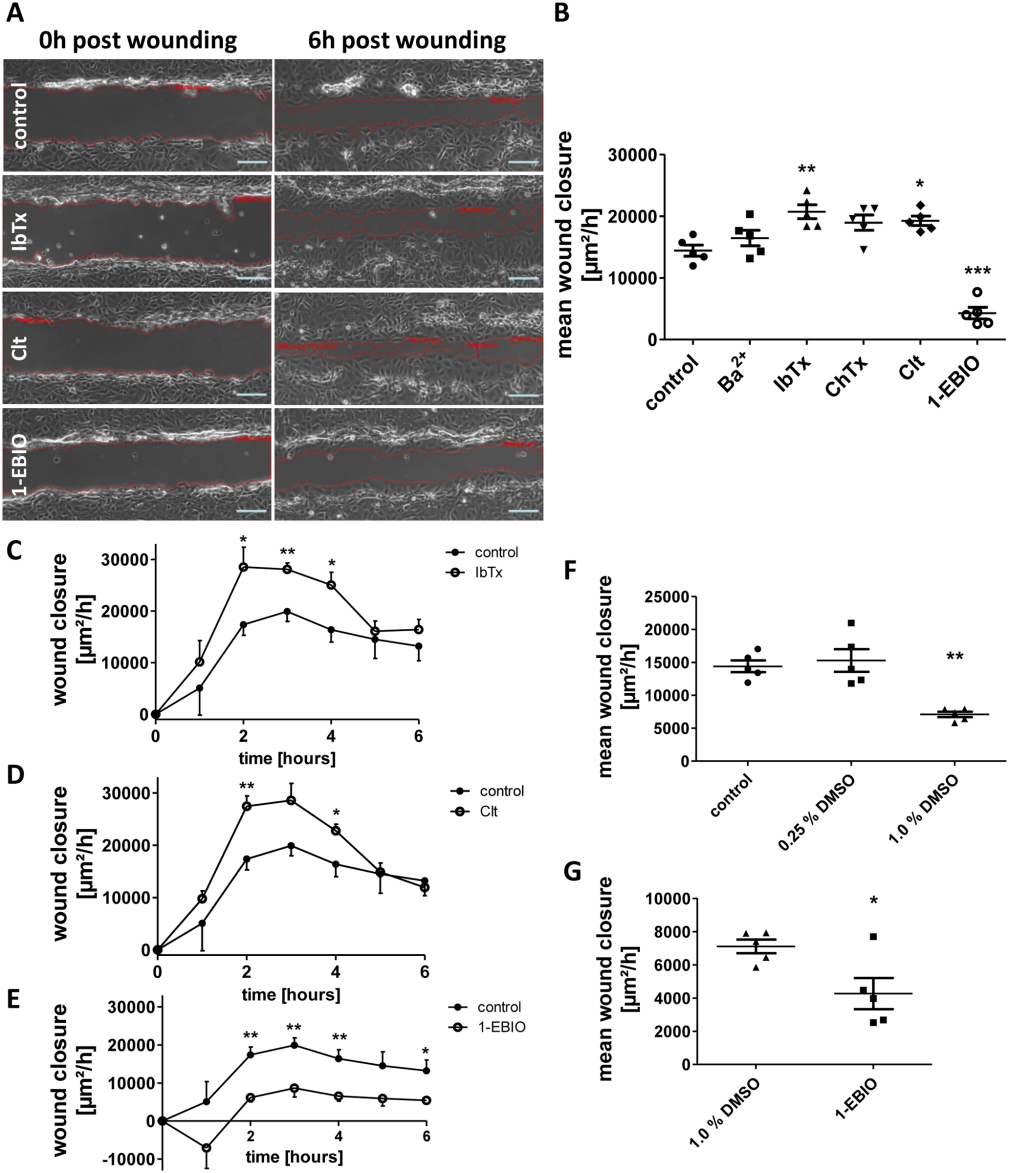
Regulation of intestinal epithelial wound healing by potassium channel modulation. Wound healing of IEC-18 within six hours after mechanical injury. A: Representative images of wounds 0 and 6 hours after wounding in the presence of the indicated potassium channel modulators. Scale bar: 100μm. B: Quantitative analysis of wound healing of IEC-18 incubated with different potassium channel modulators (n = 5). While IbTx and Clt cause an increase in intestinal epithelial wound healing response, wound closure is significantly reduced after administration of 1-EBIO. C-E: Time course of wound healing after administration of IbTx, Clt and 1-EBIO (n = 5). F: Comparison of different solvent concentration applied (n = 5). While 0.25% (v/v) DMSO does not affect wound closure, 1% (v/v) DMSO significantly retards wound closure. G: Direct comparison of 1-EBIO with its solvent (n = 5). Reduction in wound healing by 1-EBIO is also significant vs. 1% DMSO.

To test the yet barely investigated hypothesis that potassium channels might be involved in intestinal epithelial restitution as they are in the restitution of other tissues [13], [16] we performed scratch wounding assays of IEC-18 monolayers in the presence of different potassium channel modulators. Mean wound closure was determined by the area recovered by the cell sheet within six hours (Fig 2A) and was found to be significantly higher after treatment with IbTx (+43.8 +/- 7.8%) and Clt (+33.5 +/- 5.2%) than under control conditions. IbTx inhibits the Ca^2+^-dependent large-conductance potassium channel KCNMA1, while Clt inhibits the Ca^2+^-dependent intermediate-conductance potassium channel KCNN4. Addition of ChTx, which inhibits KCNMA1 and KCNN4 as well as some voltage-gated potassium channels, resulted in a close to significant effect on wound closure (+31.4 +/- 8.6%, p = 0.06). In contrast, 1-EBIO, an activator of KCNN4 and Ca^2+^-dependent small-conductance potassium channels, yielded a reduced rate of wound healing (-70.4 +/- 6.5%). Ba^2+^, which unspecifically inhibits constitutively active potassium channels, had no significant effect (Fig 2B).

Furthermore, the current rate of wound area recovered in each hour after wounding was determined. For both IbTx and Clt, enhanced wound healing was due to significantly raised healing rates during the second, third and fourth hour after wounding. The 1-EBIO dependent growth rates were reduced almost over the complete course of the experiment (Fig 2C–2E).

In order to find out, whether results could be distorted by the necessary use of DMSO as solvent for Clt (0.25%) and 1-EBIO (1%), controls were performed for 0.25% (v/v) and 1.0% (v/v) DMSO in starvation medium (Fig 2F). While 0.25% DMSO had no impact on wound closure (+6.1 +/- 11.9%, p = 0.90), 1% DMSO caused a significant impairment of wound healing (-50.7 +/- 2.9%). However, when compared to 1% DMSO, 1-EBIO still led to a reduced wound closure (-39.9 +/- 13.1%; Fig 2G) suggesting that 1-EBIO intrinsically downregulates wound healing.

### Preincubation with IFN-γ inverts effects of KCNN4 inhibition on intestinal epithelial wound healing

We sought to explore how KCNN4-dependent intestinal epithelial wound healing is regulated under inflamed conditions. Thus, IEC-18 cells were pretreated with 100 ng/mL IFN-γ 24 h before wounding and the impact of Clt and 1-EBIO was studied.

**Fig 3:**
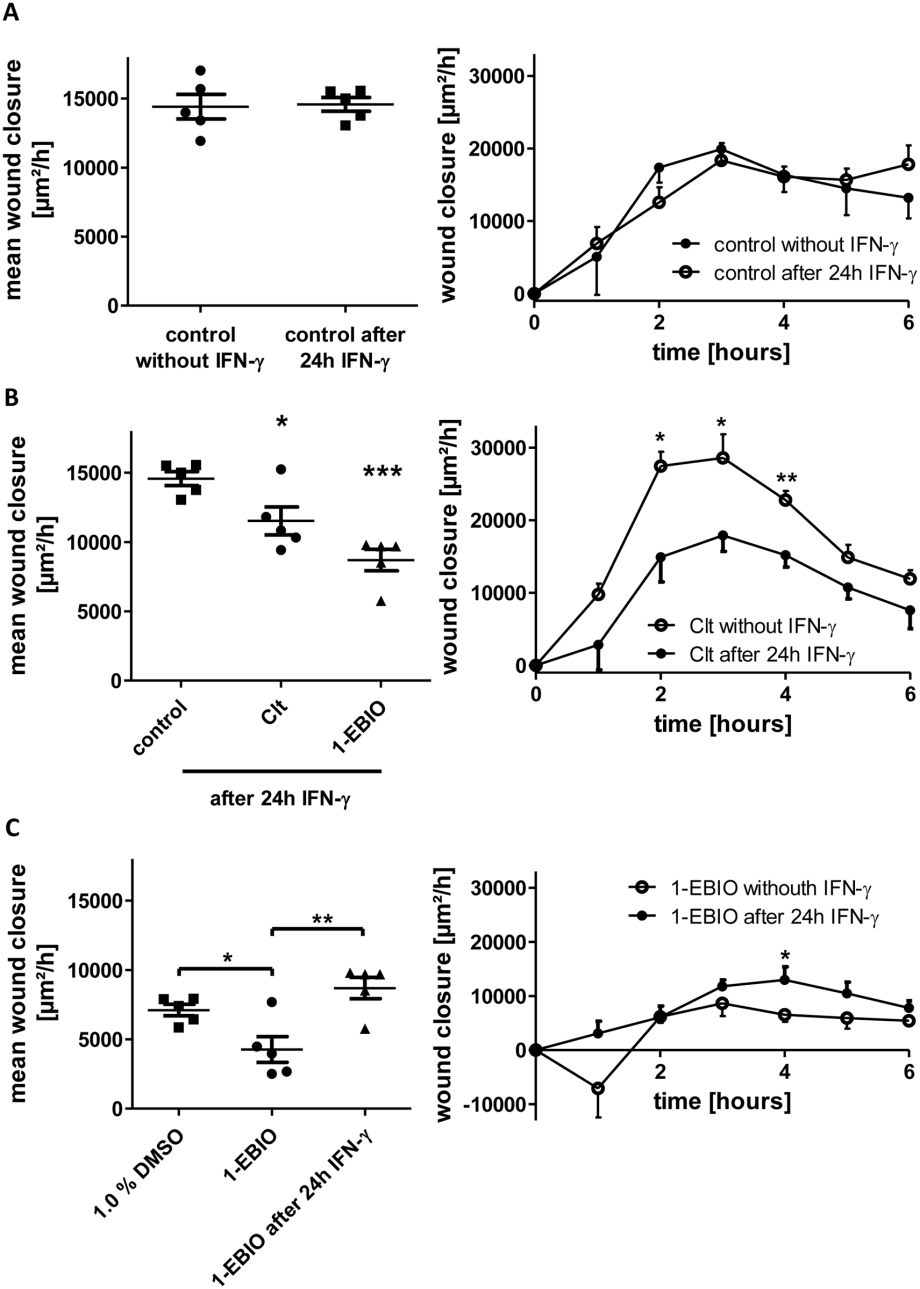
Differential regulation of KCNN4-dependent intestinal epithelial wound healing in response to preincubation with IFN-γ. Wound healing of IEC-18 within six hours after mechanical injury. A: Comparison of wound healing of IEC-18 under control conditions without and with 24 h-pretreatment with IFN-γ (n = 5). Both the mean wound closure over six hours (left panel) and the time course profile of wound healing (right panel) are similar. B: Wound healing of IEC-18 after IFN-γ pretreatment and incubation with Clt and 1-EBIO (n = 5). KCNN4-inhibition by Clt results in significantly reduced rates of wound closure (left panel). Compared to Clt treatment without IFN-γ preincubation wound closure rates upon addition of Clt after 24h IFN-γ are significantly reduced in the second, third and fourth hour (right panel). C: Comparison of 1-EBIO-dependent wound closure to 1% DMSO (left panel, n = 5) and time course of 1-EBIO-dependent restitution with and without IFN-γ preincubation (right panel, n = 5). After proinflammatory treatment, 1-EBIO-dependent wound closure is higher than under baseline conditions.

Neither overall restitution (+1.1 +/- 3.5%) nor time course of wound closure was altered by IFN-γ pretreatment under control conditions (Fig 3A). Addition of Clt exhibited a significantly reduced wound closure (-20.9 +/- 6.9%) differing from Clt-dependent healing under baseline conditions mainly in the second, third and fourth hour of the experiments. The administration of 1-EBIO also caused an impaired growth rate (-40.3 +/- 5.3%; Fig 3B).

Interestingly, the 1-EBIO-dependent wound healing rate was significantly higher than without IFN-γ pretreatment (+103.9 +/- 18.0%) and not reduced in relation to 1% DMSO alone (+22.4 +/- 10.8%; Fig 3C) indicating that the pure action of 1-EBIO might also be reversed under inflammatory conditions.

### EGF does not further increase wound healing upon KCNN4 inhibition, but completely abrogates the reversing effect of IFN-γ pretreatment on KCNN4-dependent wound closure

As EGF and corresponding signaling pathways have been shown to influence migration and wound healing in several types of epithelial cells [36], [37] we investigated the hypothesis that these could also be implicated in the potassium channel-modulated wound healing response of IEC. Therefore, 5 nM EGF were added additionally to potassium channel modulators with and without prior IFN-γ incubation.

**Fig 4:**
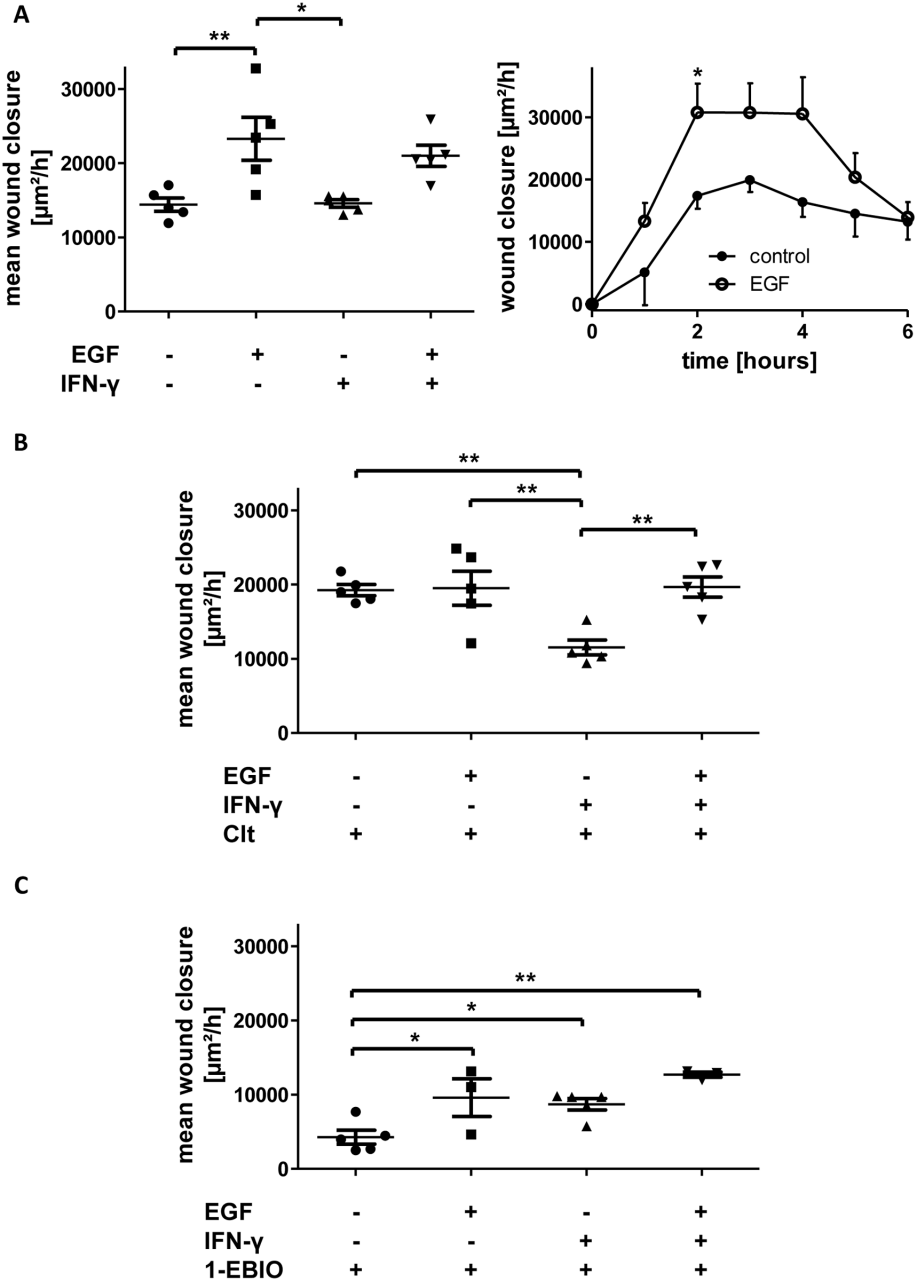
Potassium channel-dependent wound healing of IEC-18 with and without 5 nm EGF under baseline or IFN-γ pretreated conditions. Wound healing of IEC-18 within six hours after mechanical injury. A: Influence of EGF on intestinal epithelial wound healing (n = 5). EGF increases wound healing both under baseline and inflammatory conditions (left panel). Right panel: Time course of restitution upon EGF treatment compared to control showing marked differences in the second, third and fourth hour after wounding. B: Impact of Clt with and without additional EGF after or without IFN-γ pretreatment (n = 5). EGF does not further increase Clt-dependent wound healing under baseline conditions, but fully reverts the Clt-dependent reduction in wound closure after proinflammatory treatment. C: Impact of 1-EBIO with and without additional EGF after or without IFN-γ pretreatment (n = 3–5). EGF promotes 1-EBIO-mediated wound healing both under baseline and inflammatory conditions.

EGF alone significantly increased wound healing compared to control conditions (+61.5 +/- 20.1%). Following 24h IFN-γ pretreatment, rise in wound closure after EGF administration was close to significant (+44.1 +/- 9.8%, p = 0.07). Regarding the time course of wound healing, marked differences in the second, third and fourth hour after wounding were responsible for the effect of EGF (Fig 4A).

The combination of EGF with Clt (Fig 4B) did not further augment the Clt-dependent growth rate observed under baseline conditions (+1.3 +/- 11.9%). After pretreatment with IFN-γ wound closure in response to Clt together with EGF was similarly raised. Thus, the inverting effect of IFN-γ pretreatment on Clt-dependent wound healing was completely abrogated by additional administration of EGF (+70.5 +/- 11.9%).

The addition of EGF together with 1-EBIO (Fig 4C) caused significantly higher wound healing rates than measured with 1-EBIO alone (+124.8 +/- 59.8%). Preincubation with IFN-γ and subsequent treatment with 1-EBIO and EGF yielded a further, not significant, rise in wound closure as to the growth rates observed with 1-EBIO alone under inflammatory conditions (+45.8 +/- 4.2%).

We therefore conclude that accelerated wound healing after inhibition of KCNN4 could be due to an activation of signaling pathways that are also activated by binding of EGF to its receptor EGFR, because wound closure cannot be further stimulated by additional application of EGF. Consistent with that, EGF raised growth rates reduced by 1-EBIO-dependent activation of KCNN4.

Furthermore, retardation of wound healing after KCNN4 inhibition following IFN γ-pretreatment could be explained by a perturbation of this linkage to EGFR-dependent pathways by IFN-γ, as their direct activation through EGF is able to reestablish enhanced wound healing.

Of note, IbTx-dependent epithelial restitution did not differ between baseline and inflammatory conditions (S2 Fig) suggesting that different potassium channels play different functions in regulating wound healing.

### IEC migration is differentially affected by KCNN4 modulation

Although scratch assays are commonly accepted as a valid *in vitro* model of cell migration and we have shown above that proliferation does not play a relevant role within six hours after wounding, we aimed at further specific assessment of IEC migration. Thus, Boyden chamber assays were performed.

In concrete terms, cells were starved with or without 100 ng/mL IFN-γ for 24 h and allowed to migrate through a polycarbonate membrane into compartments with starvation medium or starvation medium with Clt during six hours.

Without proinflammatory pretreatment, migration was increased in response to Clt (+34.8 +/- 8.3%) vs. control conditions.

**Fig 5:**
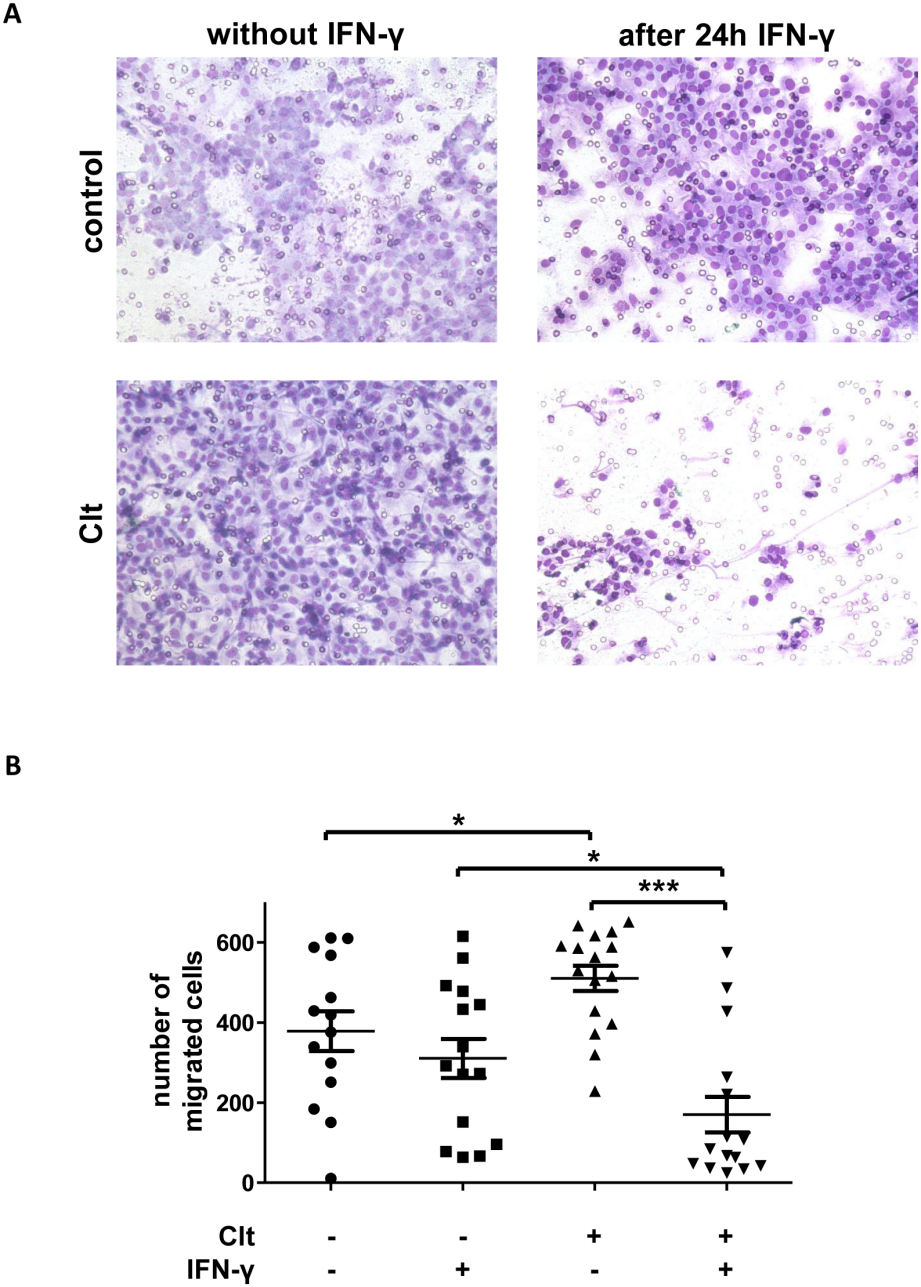
Boyden chamber results. A: Representative images of stained cells attached to the lower surface of polycarbonate membranes after migration of IEC-18 towards medium containing Clt or not and after or without IFN-γ pretreatment. B: Quantitative analysis of the number of cells migrated through a polycarbonate membrane during six hours under the indicated conditions (n = 14–16). Clt-dependent migration is significantly higher under baseline than under inflammatory conditions.

Following preincubation with IFN-γ, migration under control conditions was not altered in comparison to not pretreated controls (-18.0 +/- 12.9%). Clt (-66.7 +/- 34.7%) yielded a significantly decreased number of migrating cells vs. Clt without IFN-γ-pretreatment (Fig 5).

This finding parallels scratch assay results and therefore further supports the notion, that altered migration in response to KCNN4 modulation and IFN-γ pretreatment is a major reason for the differential regulation of wound healing observed in the scratch assays.

### Changes in PI3K- but not in MAPK-signaling pathway correlate with differential regulation of intestinal wound healing by Clt

**Fig 6:**
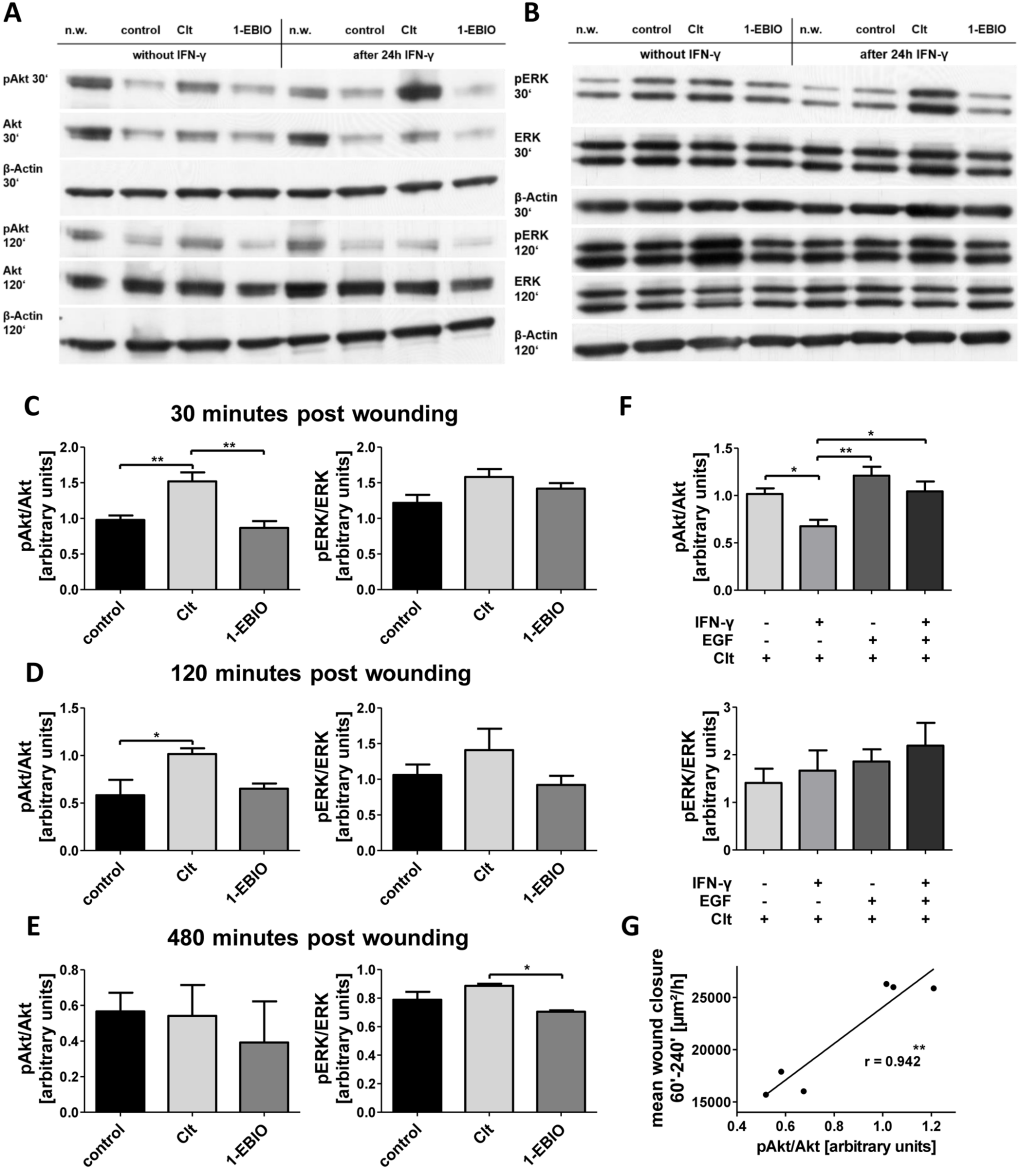
Clt-dependent alterations in Akt but not ERK phosphorylation correlate with wound healing of IEC-18. A: Representative blots showing Akt phosphorylation after 30 and 120 minutes. B: Representative blots showing ERK phosphorylation after 30 and 120 minutes. n.w.–not wounded. C-E: Quantitative analysis of the phosphorylation of Akt (left panels) and ERK (right panels) as determined by pAkt/Akt and pERK/ERK, respectively, after 30 (C), 120 (D) and 480 minutes (E). Clt enhances Akt but not ERK phosphorylation after 30 and 120 minutes. n = 3–7. F: Phosphorylation of Akt (upper panel) and ERK (lower panel) in Clt-involving conditions 120 minutes after wounding (n = 3–5). While ERK phosphorylation is not significantly altered, Akt phosphorylation is reduced by IFN-γ pretreatment and reraised by additional EGF, thus correlating Clt-dependent wound closure in scratch assays. G: Correlation of pAkt/Akt with mean wound closure in the second to fourth hour of the scratch assays in controls and Clt-involving constellations. Relative phosphorylation and wound closure rates (S1 Table) were plotted against each other and a regression line was computed. Pearson’s coefficient is 0.942 (p = 0.005).

We conducted WB analysis (Fig 6A+6B) in order to explore the hypothesis that EGFR-activated pathways might be involved in the observed differential regulation of intestinal wound healing in response to Clt treatment under inflamed conditions. Therefore, phosphorylation of Akt and ERK-1/2 as key kinases of the PI3K- and MAPK-pathway was determined in the constellations indicated above by correlation of phosphorylated protein to total protein.

Under baseline conditions (Fig 6C–6E), Akt phosphorylation was enhanced by Clt vs. control (+55.5 +/- 12.8%) and vs. 1-EBIO (+75.7 +/- 32.4%) 30 minutes after wounding. ERK phosphorylation was not altered by these potassium channel modulators.

Two hours post wounding, increased Akt phosphorylation vs. control could once more be observed following addition of Clt (+74.3 +/- 10.4%). Again, there were no differences in ERK phosphorylation.

At 480 minutes after wounding, Akt phosphorylation was on basal levels again. Clt-dependent ERK phosphorylation was slightly elevated in contrast to 1-EBIO (+25.8 +/- 3.7%).

Therefore, the Clt-dependent increase of wound closure rates was paralleled by a rise of phosphorylated Akt but not ERK after 30 and 120 minutes. We subsequently particularly focused on the WB findings 120 minutes post wounding, as time course analysis of the scratch assays had shown that differences in wound healing rates were mainly due to different growth rates in the second, third and fourth hour after wounding (Fig 2D).

In the presence of Clt (Fig 6F), IFN-γ pretreated cells displayed significantly reduced Akt phosphorylation 120min after wounding compared to not preincubated cells (-33.5 +/- 16.2%). Addition of EGF in combination with Clt under baseline conditions did not increase Akt phosphorylation vs. Clt alone. After preincubation with IFN-γ, additional EGF was able to completely abolish the effect of Clt on Akt phosphorylation as levels were significantly raised (+54.7 +/- 26.7%) and comparable to those without preincubation. ERK phosphorylation was not significantly altered for either of these conditions 120 minutes after wounding.

Thus, Akt phosphorylation after 120 minutes and wound healing growth rates showed a remarkable resemblance. To investigate this in more detail, we correlated Akt-phosphorylation and mean wound closure 60–240 minutes after wounding in controls and Clt-involving constellations. With a Pearson's coefficient of 0.942 (p = 0.005) this correlation turned out to be very strong and statistically significant (Fig 6G). Though not causally proving, this strongly argues for an involvement of the PI3K signaling cascade in the KCNN4-mediated regulation of intestinal epithelial wound healing.

### Clt-mediated increase in wound healing of HT-29 cells is abrogated by PI3K but not ERK inhibition

**Fig 7:**
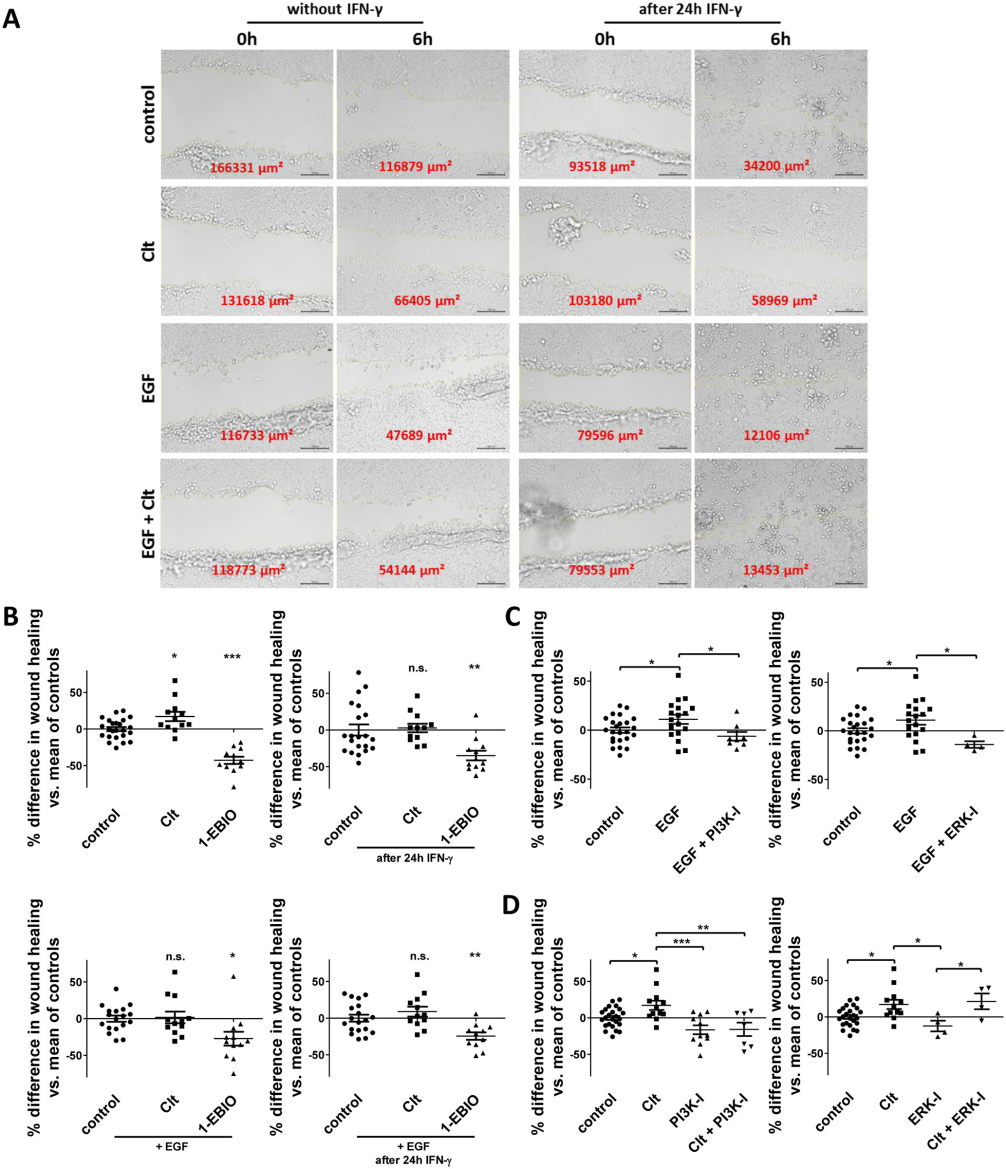
Scratch-wounding of HT-29 cells confirms a differential regulation of KCNN4-dependent restitution with crucial contribution of PI3K but not ERK signaling. Confluent monolayers of HT-29 cells were wounded as explained in the Methods section and imaged right after wounding and after 6h. A: Representative images of the wounds at the indicated time points upon treatment with the indicated compounds without (left panels) or after (right panels) 24h pretreatment with IFN-γ. Wound size is denoted in red. Scale bar: 100 μm. B: Quantitative analysis of wound healing under baseline conditions (left panels) or with proinflammatory treatment (right panels) and without (upper panels) or with EGF treatment (lower panels), respectively (n = 11–24). Individual wound closure is displayed as % difference vs. the mean of respective controls. Clt increases wound healing vs. controls in baseline but not inflammatory conditions. Clt in combination with EGF does not further increase EGF-dependent wound healing. C: Impact of PI3K (left panel) and ERK inhibition (right panel) on EGF-dependent intestinal epithelial restitution (n = 4–24). Both compounds revert the increase observed with EGF. D: Impact of PI3K (left panel) and ERK inhibition (right panel) on Clt-dependent intestinal epithelial wound healing (n = 4–24). The Clt-mediated increase in wound closure is abrogated by addition of the PI3K inhibitor but not the ERK inhibitor.

In an attempt to confirm and extend our results we performed an additional series of experiments with the human intestinal epithelial cell line HT-29 (Fig 7A). Again, Clt increased wound healing vs. controls in baseline but not in inflammatory conditions, while EGF did not further increase Clt-mediated wound healing (Fig 7B). Once more, 1-EBIO-dependent restitution was lowered (S4 Fig).

Furthermore, HT-29 were additionally treated with the PI3K inhibitor Ly294002 or the ERK inhibitor 3-(2-Aminoethyl)-5-((4-ethoxyphenyl)methylene)-2,4-thiazolidinedione-hydrochloride. Both compounds were able to reduce the increased wound healing observed upon EGF treatment back to baseline (Fig 7C). Finally, when cells were treated with Clt and one of the inhibitors, Clt-mediated increase in restitution was unaffected by ERK inhibition but significantly reduced by PI3K inhibition (Fig 7D) further backing our hypothesis of a crucial role of the PI3K cascade in the mediation of KCNN4-dependent effects on intestinal epithelial restitution.

### mRNA-levels of KCNN4 are augmented in IBD

Aiming to assess a possible role of KCNN4 in human IBD, we isolated IEC from surgical specimens and endoscopic biopsies and quantified the expression of KCNN4 mRNA by TaqMan^®^ Real Time PCR.

**Fig 8:**
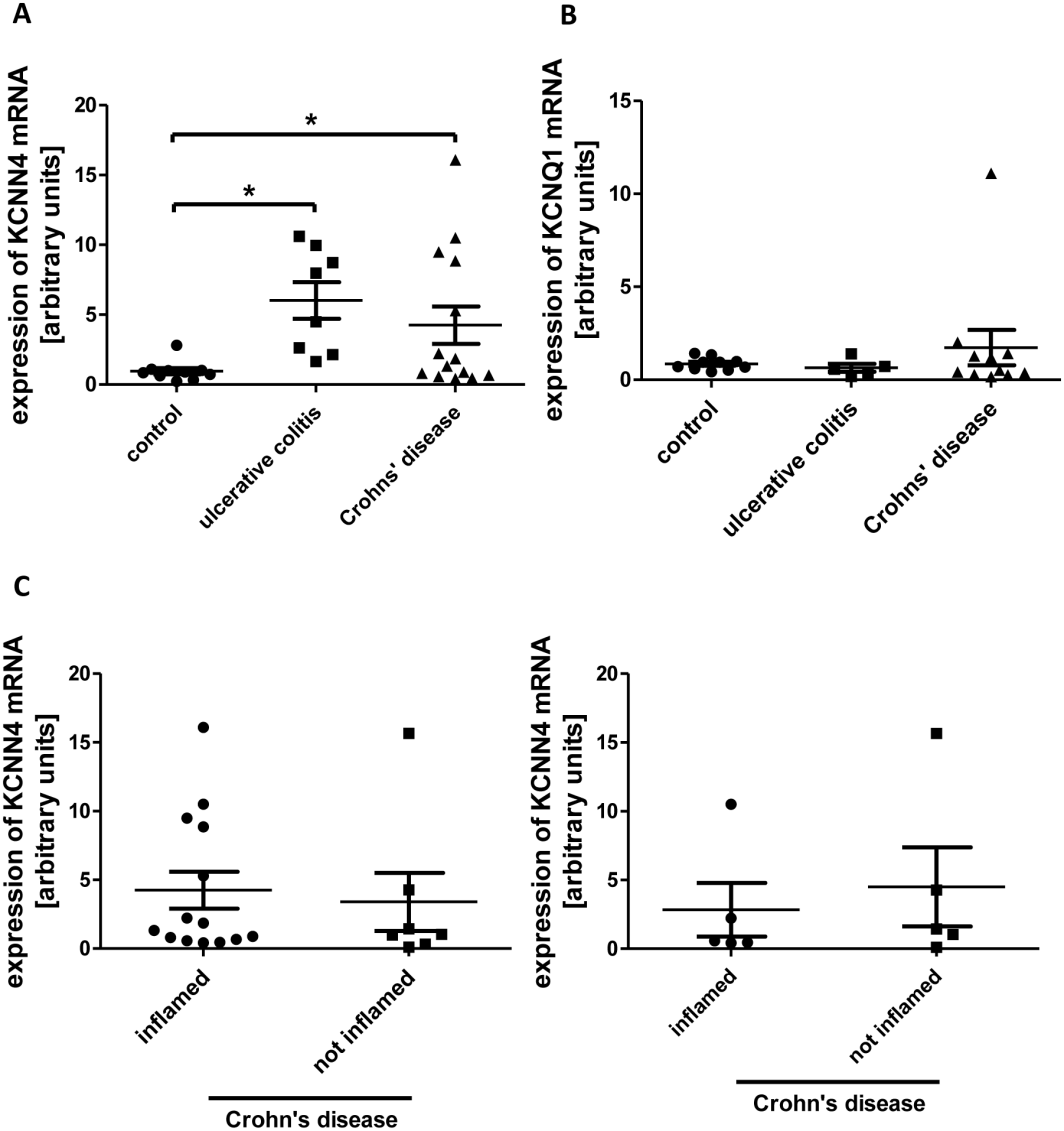
KCNN4 mRNA expression in IBD. A: Relative levels of KCNN4 mRNA in IEC from controls (n = 10), UC (n = 8) and CD (n = 14). In both UC and CD the expression is increased vs. control. B: KCNQ1 mRNA levels are equal in UC (n = 5), CD (n = 11) and controls (n = 10) C: Comparison of KCNN4 expression in inflamed vs. uninflamed tissue of patients with CD. Left panel: Relative KCNN4 mRNA levels in all available samples (n = 14 inflamed, n = 7 not inflamed). Right panel: Comparison of KCNN4 levels in inflamed and uninflamed samples originating from the same patients (n = 5).

In cells from macroscopically inflamed tissue from both UC (+531.5% +/- 137.2%; n = 8) and CD patients (+345.7% +/- 140.4%; n = 14) the expression was increased in comparison to control cells (n = 10), which were collected from macroscopically normal mucosa of patients who underwent operation because of CRC or SD (Fig 8A). Moreover, mRNA-expression of KCNQ1, a voltage-gated potassium channel, was determined in most of the samples to exclude an unspecific effect on potassium channels, but in no condition expression of KCNQ1 mRNA was significantly altered (Fig 8B).

We were also able to collect some specimens from macroscopically unaffected gut tissue of CD patients (as determined by an independent pathologist) and compared KCNN4 mRNA expression in these samples to those from inflamed tissue (Fig 8C). No significant difference could be revealed both when comparing all samples and only those where inflamed and uninflamed samples came from the same patient.

## Discussion

The importance of intestinal epithelial wound healing for sustaining an intact intestinal barrier in the context of inflammation has been emphasized by many authors [2], [20], [38]. Although there is a large body of research suggesting an important role for potassium channels in wound healing and migration, their role in intestinal epithelial restitution is poorly defined. The same applies for IFN-γ, which has been shown to impair many cellular functions [18], [25], [26], [39], but up to now, its implications for wound healing are not completely understood.

Using the rat-derived non-transformed IEC line IEC-18 [28] in scratch assays as a well-established model for migration-dominated IEC restitution [10], [15], [32], we showed that inhibition of the Ca^2+^-dependent potassium channels KCNN4 by Clt and KCNMA1 by IbTx caused a significant increase in wound healing response after mechanical injury. In contrast, activation of KCNN4 by 1-EBIO retarded wound closure. Modulation of constitutively active (e.g. voltage-gated or inwardly rectifying) potassium channels by administration of Ba^2+^ had no effect. Further experiments with the human intestinal epithelial cell line HT-29 confirmed the effects observed with both Clt and 1-EBIO.

These modulators have been extensively used to study potassium channel effects in the literature. IbTx is a highly selective toxin with no known off-target effects [40]. Barium unspecifically inhibits a range of potassium channels through blockade of the K^+^ selectivity filter as the size of not hydrated Ba^2+^ and K^+^ ions are similar, but Ba^2+^ binds more tightly due to its charge [41]. Clt is a specific inhibitor of the Ca^2+^-dependent intermediate conductance channel KCNN4 [33]. Although it has to be mentioned that Clt also inhibits cytochromes of the P450 family [42], this activity does not seem to play a role in the current study because cytochrome inhibition brings along metabolic changes which would show their effects with some temporal delay. To the contrary, epithelial restitution apparently happened shortly after wounding. Moreover, this limitation is shared by alternative KCNN4 blockers [43]. 1-EBIO has been shown to increase KCNN4 activity. However, it is also known that it activates the small conductance channels KCNN1-3 [44] and may target other molecules like adenylyl cyclase or CFTR as well [34]. Thus, the corresponding results have to be interpreted more cautiously.

An increase in wound healing following KCNN4-inhibition by Clt was reported previously in the human colon cancer-derived cell lines T84 and CaCo2, whereas the inhibitory effect of 1-EBIO could not be demonstrated in this study [14] using a comparatively low concentration [34], [45]. Interestingly, hyperpolarization of cells by potassium-free medium and thereby mimicking the effect of a potassium channel activator did impair wound healing [30].

Both IbTx and Clt as well as treatment with EGF exhibited significantly increased restitution rates in the second to fourth hour of the scratch assays. These effects early after wounding elicited the question whether the addition of potassium channel modulators might provoke the activation of intracellular signaling events to contribute to their impact on restitution. Signaling pathways like the PI3K cascade haven been identified as important mediators of intestinal epithelial wound healing and signaling molecule activation in response to appropriate stimuli was demonstrated to be present early after wounding for up to five hours [10], [46], [47]. Interestingly, the addition of EGF, which among others activates PI3K and ERK signaling cascades, not only led to time-course profiles of wound closure comparable to those in response to IbTx and Clt, but could also not further increase wound closure when added to Clt or IbTx. Thus, a transactivation of EGFR-dependent signaling pathways by potassium channel inhibition might account for these observations.

An interaction of different potassium channels with signaling molecules and especially growth factor receptors or their downstream signaling pathways has already been demonstrated in several other cell types [48]. For example, inhibition of the channel KCNH2 in leukemic cells blocked migration through a complex with VEGFR and ß1-integrin [49]. In human T-cells, KCNA3 formed a complex with ß1-integrin, which seems to be implicated in propagation and retardation of migration by activation or inhibition of the channel, respectively [50], [51]. KCNMA1 has been shown to directly interact with FAK in human osteoblasts [52]. Besides these examples for conformational coupling, an interaction with signaling molecules through changes of the membrane potential or ion flux is possible [53].

Another potential mechanism how potassium channels influence restitution is based on polar differentiation of cells during migration: In migrating MDCK-F-cells KCNN4 concentration was higher in the leading edge than in the rear pole due to endocytotic recycling and microtubular transport [54]. As Ca^2+^-concentration is reduced in the leading edge of migrating cells [55], this physiologically results in a high concentration of inactive channels there. Pharmacologic inhibition of KCNN4 could have an additive effect on this situation and thereby promote migration.

These observations are in contrast to studies demonstrating that KCNN4-inhibition impedes migration of multiple cell lines such as dendritic lung cells [16], MDCK-F-cells [56] and glioblastoma cells [57]. As an activation of KCNN4 in the rear pole of migrating cells is also essential for migration [58] and goes along with increased Ca^2+^-concentration [55], a cell-specific importance of these mechanisms at the front and in the back could be hypothesized. Furthermore, galvanotaxis has been proposed to account for the different effects observed in response to KCNN4-inhibition in different tissues [13].

Though the limitations mentioned above have to be kept in mind, the observations made for 1-EBIO further support a substantial role for KCNN4 in the regulation of intestinal epithelial wound healing, as the activation of KCNN4 by 1-EBIO caused the opposite effect of its inhibition by Clt and additional EGF was able to reduce the impairment of wound healing exerted by 1-EBIO alone. When compared to control with 1.0% DMSO, EGF even completely abolished this impairment.

Inflammatory conditions were mimicked by 24 h pretreatment of IEC-18 with IFN-γ as described elsewhere [25]. Following KCNN4 inhibition by Clt wound healing was significantly reduced in IEC-18 and not affected in HT-29, therefore exhibiting the opposite effect of KCNN4 blockade under baseline conditions. 1-EBIO-dependent wound healing was still significantly reduced, but was significantly higher than without IFN-γ preincubation. Compared to control with 1.0% DMSO it was even restored to normal levels, thereby endorsing the assumption of reversed action of KCNN4 on IEC migration in inflammation.

Boyden chamber assays, a widely employed method to specifically determine migration [59], confirmed the significant difference of IEC-18 migration in response to Clt between baseline and inflammatory conditions. In synopsis with our data on events at the wound margin and distant locations, this suggests that the scratch assay results also mainly reflect migration and proliferation does not play a substantial role during the relatively short timeframe of the experiments.

Additional application of EGF under inflamed conditions inverted Clt-mediated reduction in wound closure to increased wound healing on levels comparable to Clt-dependent migration without preincubation. This further supports the hypothesis of EGFR-activated pathways being involved in the influence of KCNN4 on intestinal epithelial restitution: Reduced migration after IFN-γ pretreatment and KCNN4 inhibition is accompanied by a re-established response of EGFR-depending pathways to EGF. Thus, it can be supposed that proinflammatory treatment interferes with the suggested transactivation of signaling cascades originating from EGFR.

WB analysis of Akt and ERK phosphorylation provided further evidence for this assumption: Akt phosphorylation two hours post wounding paralleled Clt-dependent wound healing in the main restitution period 60–240 minutes after wounding with a very strong correlation (r = 0.942). Regarding the deductions made from the scratch assay experiments, it is very likely that Akt as key kinase of the PI3K-pathway is transactivated by KCNN4-inhibition and thereby at least in part accounts for the observations made. This transactivation could happen either via EGFR, any other step of the cascade or directly. Indirect mechanisms of PI3K/Akt activation in IEC have been demonstrated in migration [10] as well as in mitogenesis [60] and epithelial secretion [61].

To the contrary, ERK phosphorylation was not affected in this study, which is in line with previous results, where we could show that IFN-γ pretreatment of IEC leads to a preferential activation of the PI3K signaling cascade compared to the ERK-MAPK-pathway [25].

**Fig 9:**
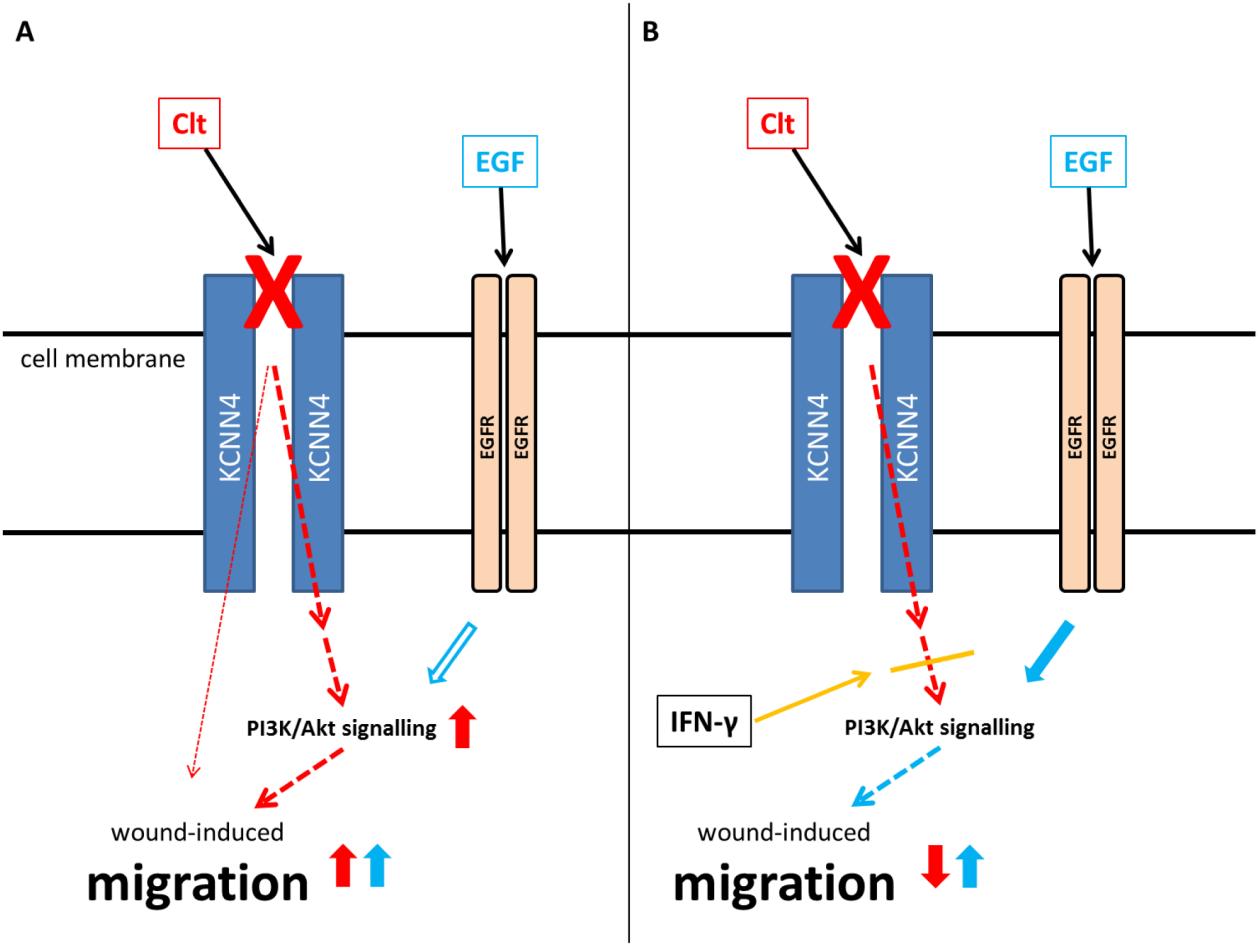
Hypothesized impact of KCNN4 inhibition on wound-induced migration and differential regulation by IFN-γ. A: Blockade of KCNN4 by Clt (red X) accelerates wound healing at least in part by transactivation of the PI3K pathway (red arrows). Additional EGF (blue) meets activated downstream signaling and exerts no additional effect (blue arrows). B: IFN-γ preincubation uncouples the postulated transactivation of PI3K signaling by KCNN4 inhibition (orange line) leading to reduced migration upon KCNN4 blockade by Clt (red X). In this constellation EGF (blue) may activate downstream targets and enhance migration (blue arrows).

IFN-γ pretreatment seems to disrupt the hypothesized transactivation cascade, therefore leading to reduced Akt phosphorylation and cell migration in response to Clt. The model presented in Fig 9 can be deduced from all these observations: Clt-mediated KCNN4 inhibition enhances wound healing at least in part by transactivating PI3K signaling (Fig 9A, red) resulting in the inability of EGF to further increase restitution (blue). On the other hand, IFN-γ pretreatment prevents KCNN4-induced PI3K signaling (Fig 9B, orange and red) and EGF is able to increase wound healing as the PI3K effector cascade is not yet activated (blue).

Reduced migration observed with 1-EBIO was not paralleled by reduced Akt-phosphorylation. We believe that this is might be due to other known effects of 1-EBIO like activatory action on other potassium channels [44] and additional impacts on other intracellular targets like the adenylyl cyclase or CFTR [34].

When interpreting these results, it has to be considered that cells not involved in wound healing were present in WB analyses and might have affected the signal-to-noise-ratio. Moreover, it has to be mentioned that these data do not prove a causal relationship underlying the demonstrated correlation of Akt phosphorylation and wound healing. However, it is a strong indication and additional scratch-wounding assays in HT-29 cells with Clt treatment in combination with PI3K and ERK inhibition could demonstrate a functional relevance of PI3K for Clt-mediated increase in wound closure as PI3K but not ERK inhibition abrogated this increase. Collectively, these observations suggest a prominent role of PI3K for KCNN4-mediated regulation of intestinal epithelial wound healing.

This is in line with former observations that the PI3K pathway essentially contributes to regulation of migration in different contexts. Karrasch et al. showed that its inhibition impairs wound healing by over 50% and that it is indispensable for wound-induced phosphorylation of GSK-3ß [10]. EGF boosts wound healing of murine colonic epithelial cells through translocation of Rac to the wound edge in a Src- and PI3K-dependent mechanism [36]. Pharmacologic activation of the PI3K-pathway by inhibition of PTEN leads to increased wound healing in corneal epithelial cells [62] and it is essential for the Gab2-mediated migration of ovarian cancer cells [63] and melanoma cells [64]. Moreover, the PI3K-cascade is more active at the leading edge of migrating cells, but is inhibited in the rear pole by PTEN [65].

Levels of KCNN4 mRNA were significantly raised in epithelium from patients suffering from CD or UC compared to patients with CRC and SD, pointing out that KCNN4 is also of possible clinical relevance. The nature of this relevance remains an object of speculation for the moment. But given our results it can be conjectured that expression of KCNN4 in IEC of IBD patients with active disease is raised reactively in order to promote wound healing. Other reports favoring an important role of KCNN4 in IBD come from Ayabe et al., who showed that inhibition of KCNN4 reduces the secretion of α-defensins [66], and from Simms et al., who demonstrated an association between a single nucleotide polymorphism in KCNN4 and ileal CD [67].

Al-Hazza et al., however, report of a decrease in KCNN4 activity in IEC of patients with active UC and postulate that reduced salt and water absorption might contribute to diarrhea [68]. However, this in in contradiction to data showing that Clt ameliorates diarrhea in infectious colitis models [69].

Recently, two studies with rodent models of colitis reported a decrease in disease severity after KCNN4 inhibition [70], [71], which seems to contradict our hypothesis of beneficial effects of KCNN4 activation on mucosal healing under inflammatory conditions. However, in both settings, lymphocytes and/or macrophages were assumed to be the main targets of KCNN4 inhibition and the role of IEC was not directly addressed.

It can be concluded, that although available experimental data fails to provide a conclusive conception of the role of KCNN4, it appears to play an important part in the pathogenesis of IBD and emerges as a potential future therapeutic target.

Taken together, in our cellular model inhibition of the potassium channel KCNN4 in the absence of any further stimulus promotes wound healing. To the contrary, mimicking inflammatory conditions with IFN-γ pretreatment leads to reduced or unaffected restitution rates after inhibition of KCNN4. Activation of EGFR by EGF in inflammatory conditions reverses this finding. A transactivation of the PI3K pathway is the presumptive mediator of these effects.

Therefore, we show–to our knowledge for the first time–that intestinal epithelial restitution is differentially regulated by specific potassium channel modulation. This reverse regulation could possibly promote or maintain chronic inflammation as it occurs in IBD.

## Supporting Information

S1 Fig Proliferation does not relevantly contribute to intestinal restitution in the observed timeframe. A: All IEC-18 cells present on hourly images of a representative scratch-wounding experiment were edged in red and counted. As displayed, the total number of cells does not substantially change, suggesting that proliferation does not relevantly contribute to intestinal restitution within the observed timeframe. B: Cell density at the wound margin and at distant locations was assessed by defining representative areas of interest at respective sites and counting the number of containing cells every hour from 0 to 6 hours. Upper panels show representative countings at 0h (left) and 6h (right) as indicated. Lower panel: Quantitative analysis of cell density at the wound margin and at distant locations. Density is normalized to initial cell number. While cell density in the distance does not relevantly change, cell density at the wound margin significantly decreases over the course of the experiments (n = 4). Asterisks indicate significant differences vs. initial cell density.
(TIF)

S2 Fig Potassium channel-dependent wound healing of IEC-18 with and without 5 nm EGF under baseline or IFN-γ pretreated conditions. Wound healing of IEC-18 within six hours after mechanical injury. A: Impact of IbTx with and without additional EGF after or without IFN-γ pretreatment (n = 3–5). No significant changes in intestinal epithelial wound healing response can be observed. B: Synopsis of intestinal epithelial restitution with or without different potassium channel modulators and/or EGF after or without IFN-γ pretreatment (n = 3–5). Constellations involving the same potassium channel modulator are connected by dashed lines. For better readability indication of significances is omitted but can be seen in Fig 4B + 4C.
(TIF)

S3 Fig Clt-dependent alterations in Akt but not ERK phosphorylation correlate with wound healing of IEC-18. A: Uncropped blots from which the lanes shown in Fig 6A and 6B derive. Note: For parallel processing with different antibodies membranes were cut in pieces at the dashed lines and later reassembled for developing. Exposure time was optimized for the indicated bands and quantification was only performed for these. B: Akt (left panel) and ERK phosphorylation (right panel) without potassium channel modulation under baseline and inflammatory conditions with or without additional EGF (n = 3–5).
(TIF)

S4 Fig Influence of DMSO on wound healing in HT-29 cells. Left panel: Impact of different concentrations of the solvent DMSO on epithelial restitution (n = 12–24). While 0.25% DMSO has no effect on wound closure, 1.0% DMSO leads to significantly reduced wound healing. Right panel: Direct comparison of 1-EBIO with its solvent (n = 12). Reduction in wound closure by 1-EBIO is also significant vs. 1.0% DMSO.
(TIF)

S1 Table Values used for Fig 6G. (TIF)

S1 Video Restitution of scratch-wounded IEC-18 monolayers. A confluent IEC-18 monolayer was mechanically wounded and imaged every 15 minutes for six hours. A video sequence was composed using MAGIX Video (Berlin, Germany) and is repeated several times to demonstrate different issues. A: Representative time lapse video of epithelial restitution. B: Cells located at the wound margin migrate into the denuded area protruding filopodia (red arrows) and pseudopodia (orange arrows). C: Five representative cells directly adjacent to the wound are edged in red. They migrate into the denuded area undergoing profound morphological changes. D: Five representative cells closely but not directly neighboring the wound are edged in red. They also engage in wound closure by migrating behind the first cell line, therefore showing properties of ‘collective sheet migration’. E: Five representative cells distant from the wound are edged in red. Over the time, they barely move nor do they considerably change shape.
(MP4)

## Abbreviations

1-EBIO: 1-ethyl-2-benzimidazolinone
BCA: bicinchoninic acid
CD: Crohn's disease
cf.: confer
ChTx: charybdotoxin
Clt: clotrimazol
CRC: colorectal carcinoma
ECL: enhanced chemiluminescence
EGFR: epidermal growth factor receptor
IBD: inflammatory bowel disease
IbTx: iberiotoxin
IEC: intestinal epithelial cells
IFN-γ: interferon-gamma
PI3K: phosphoinositide 3-kinase
SD: sigma diverticulitis
TBST: tris buffered saline with 0.1% Tween 20
TGF-ß: transforming growth factor ß
Th1: T-helper cell 1
UC: ulcerative colitis
WB: Western Blot
