# Supplementary figures and images for: K+ Channel Inhibition Differentially Regulates Migration of Intestinal Epithelial Cells in Inflamed vs. Non-Inflamed Conditions in a PI3K/Akt-Mediated Manner
Source: PLoS One. 2016 Jan 29;11(1):e0147736. doi: 10.1371/journal.pone.0147736 (PMC4732808; doi:10.1371/journal.pone.0147736)

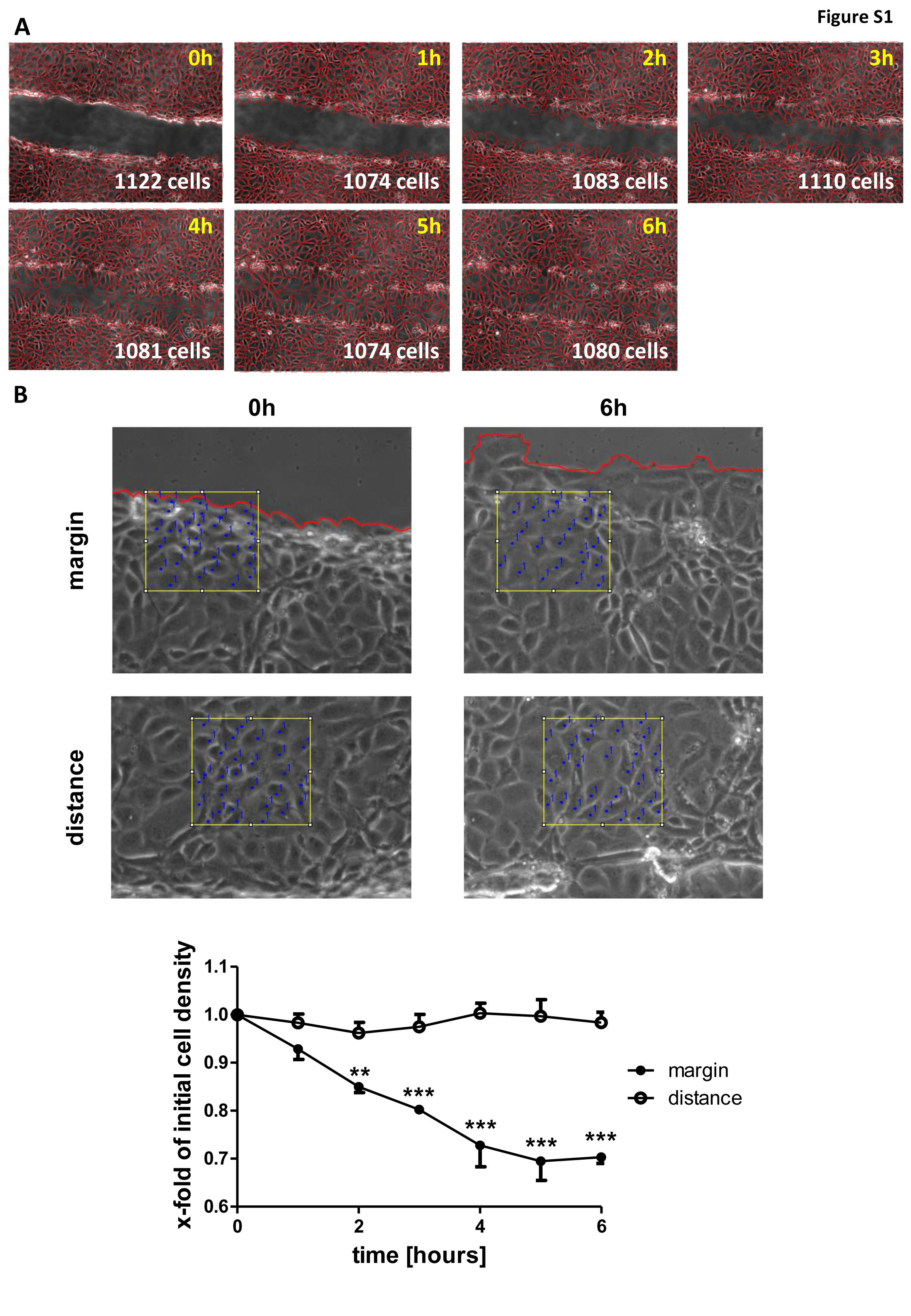

Supplement: S1 Fig — A: All IEC-18 cells present on hourly images of a representative scratch-wounding experiment were edged in red and counted. As displayed, the total number of cells does not substantially change, suggesting that proliferation does not relevantly contribute to intestinal restitution within the observed timeframe. B: Cell density at the wound margin and at distant locations was assessed by defining representative areas of interest at respective sites and counting the number of containing cells every hour from 0 to 6 hours. Upper panels show representative countings at 0h (left) and 6h (right) as indicated. Lower panel: Quantitative analysis of cell density at the wound margin and at distant locations. Density is normalized to initial cell number. While cell density in the distance does not relevantly change, cell density at the wound margin significantly decreases over the course of the experiments (n = 4). Asterisks indicate significant differences vs. initial cell density. (TIF) [file pone.0147736.s001.tif]

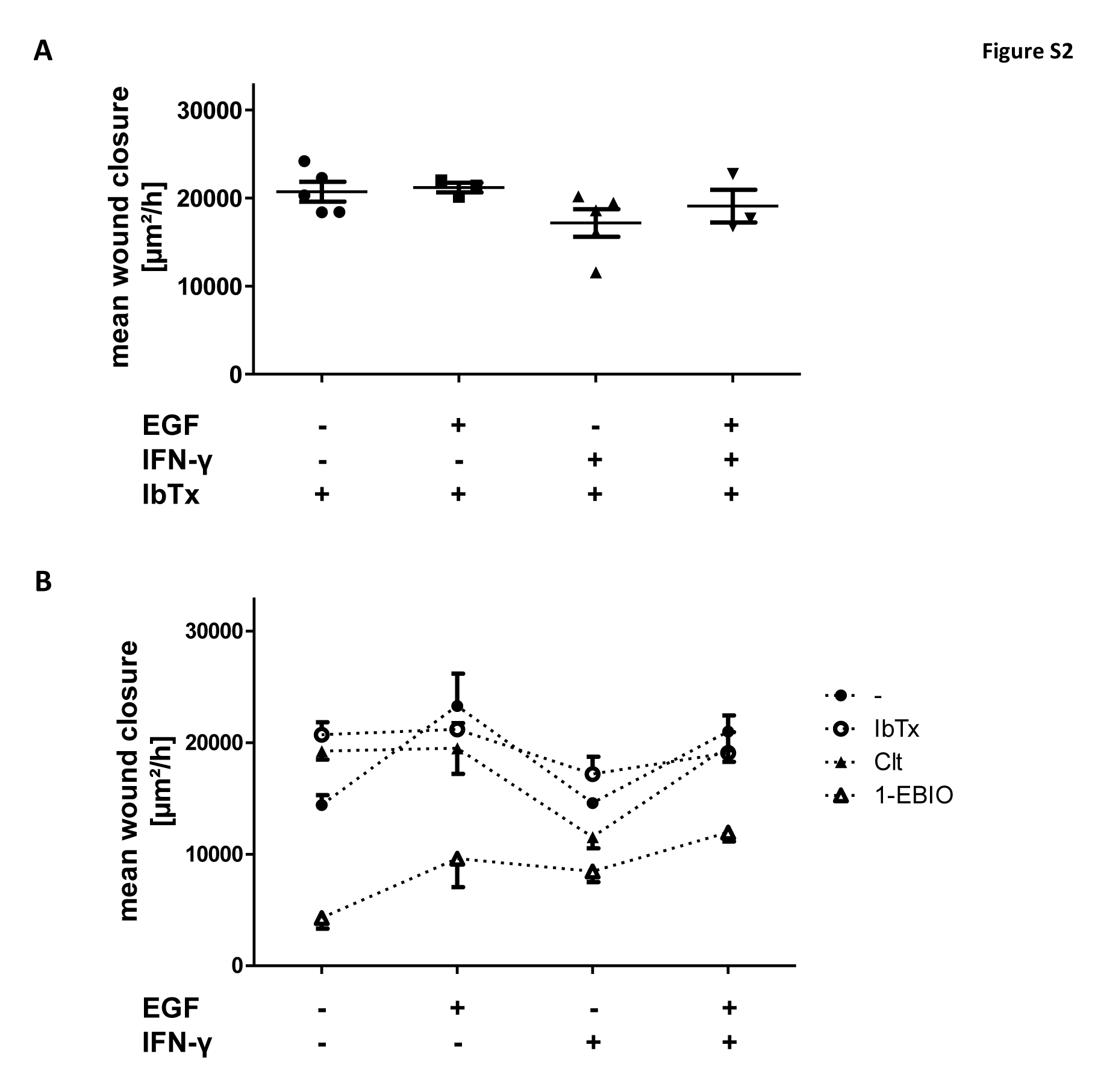

Supplement: S2 Fig — Wound healing of IEC-18 within six hours after mechanical injury. A: Impact of IbTx with and without additional EGF after or without IFN-γ pretreatment (n = 3–5). No significant changes in intestinal epithelial wound healing response can be observed. B: Synopsis of intestinal epithelial restitution with or without different potassium channel modulators and/or EGF after or without IFN-γ pretreatment (n = 3–5). Constellations involving the same potassium channel modulator are connected by dashed lines. For better readability indication of significances is omitted but can be seen in Fig 4B + 4C. (TIF) [file pone.0147736.s002.tif]

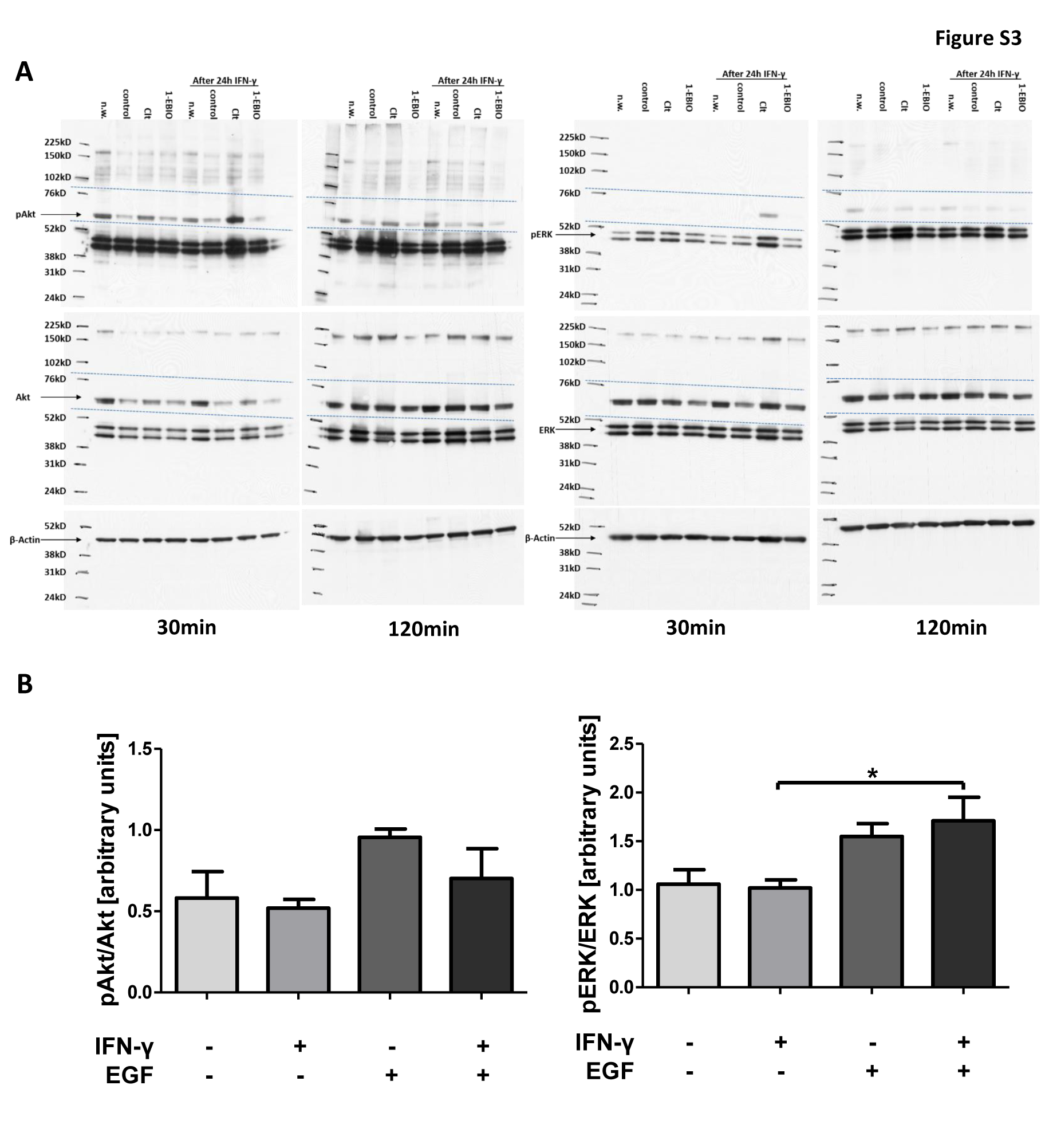

Supplement: S3 Fig — A: Uncropped blots from which the lanes shown in Fig 6A and 6B derive. Note: For parallel processing with different antibodies membranes were cut in pieces at the dashed lines and later reassembled for developing. Exposure time was optimized for the indicated bands and quantification was only performed for these. B: Akt (left panel) and ERK phosphorylation (right panel) without potassium channel modulation under baseline and inflammatory conditions with or without additional EGF (n = 3–5). (TIF) [file pone.0147736.s003.tif]

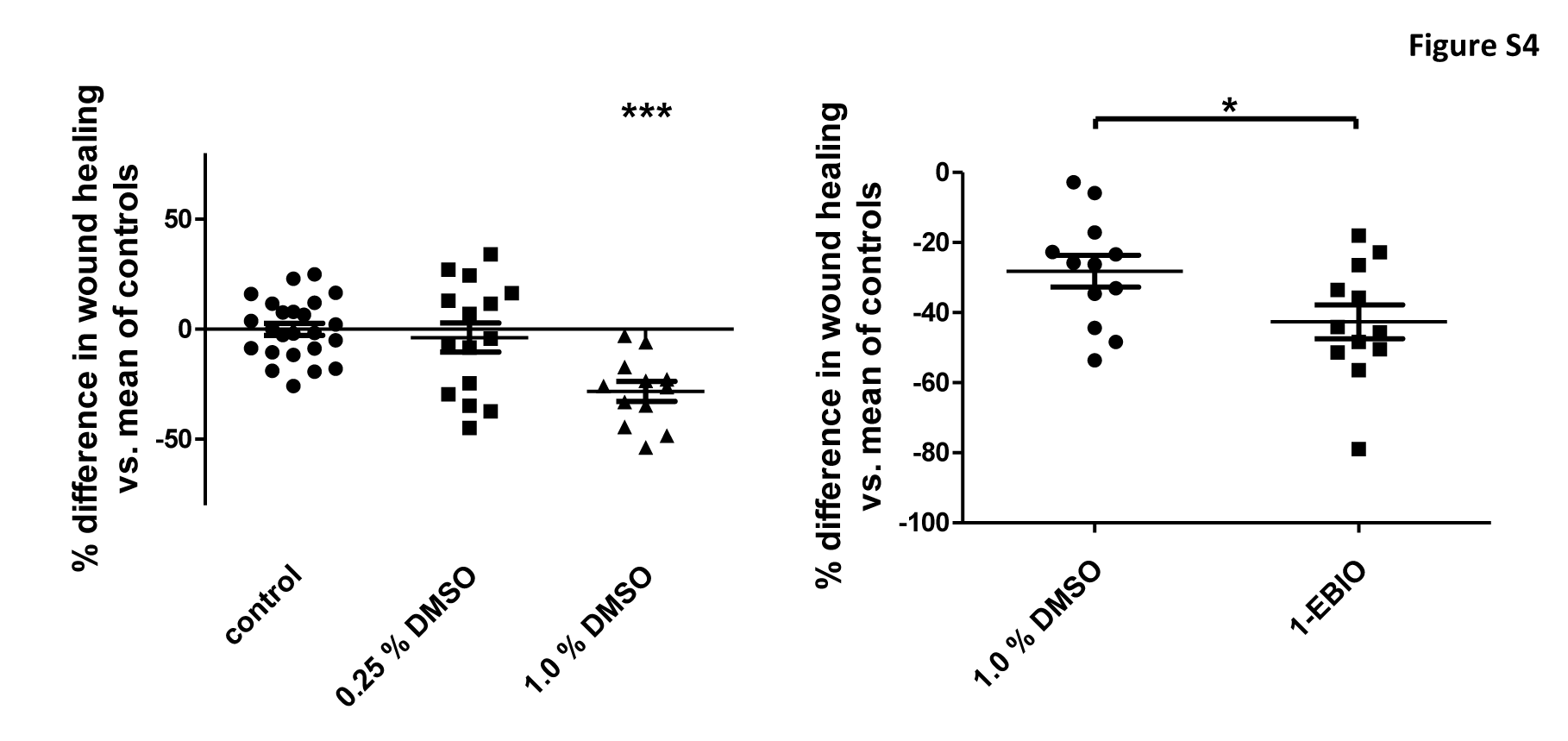

Supplement: S4 Fig — Left panel: Impact of different concentrations of the solvent DMSO on epithelial restitution (n = 12–24). While 0.25% DMSO has no effect on wound closure, 1.0% DMSO leads to significantly reduced wound healing. Right panel: Direct comparison of 1-EBIO with its solvent (n = 12). Reduction in wound closure by 1-EBIO is also significant vs. 1.0% DMSO. (TIF) [file pone.0147736.s004.tif]

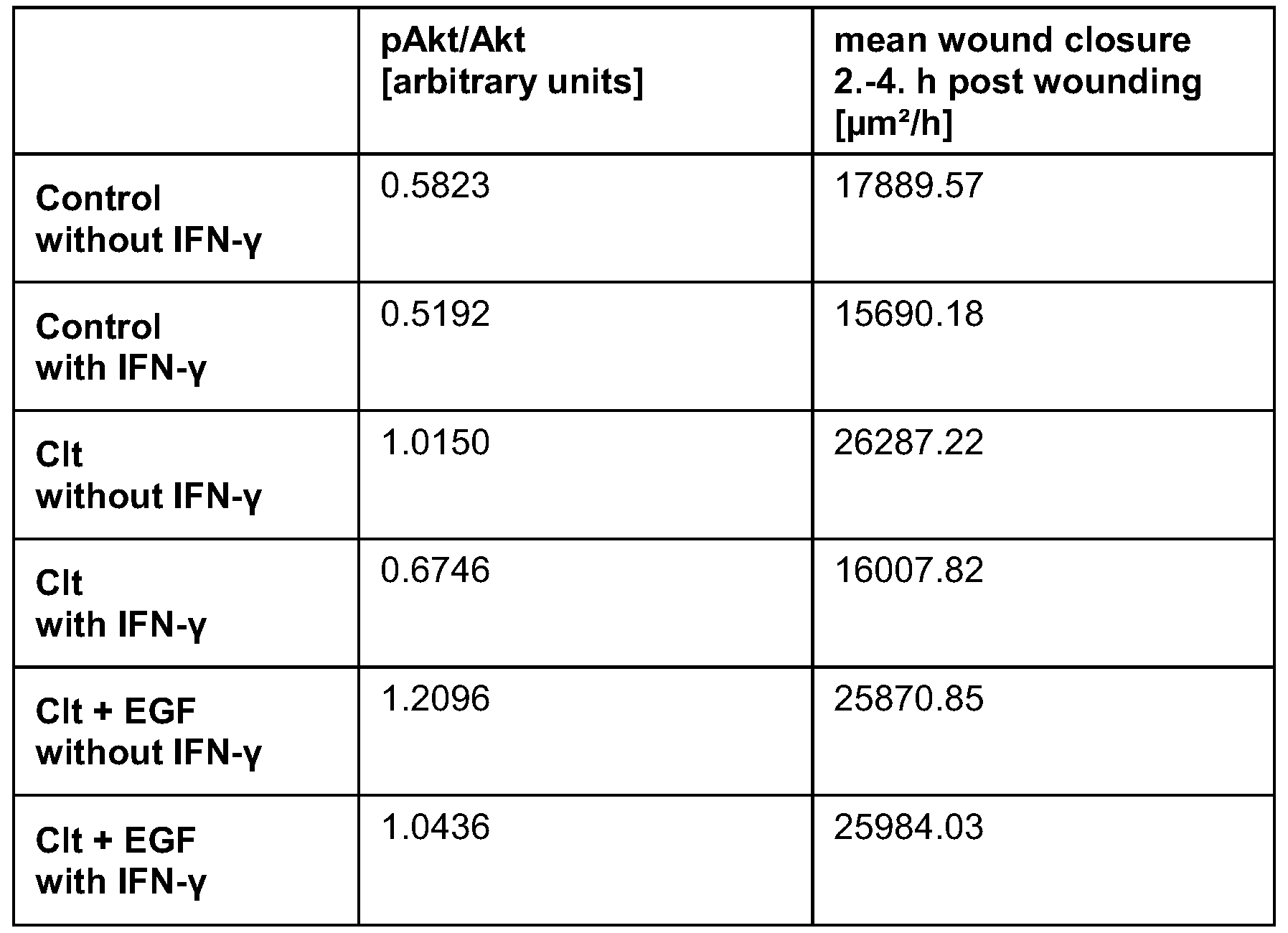

Supplement: S1 Table — (TIF) [file pone.0147736.s005.tif]
